# Bioreactor-based production of eumelanin nanoparticles by *Streptomyces glaucescens* NEAE-H: physicochemical characterization, anticancer and anti-inflammatory activities, and in silico analysis

**DOI:** 10.1038/s41598-026-65150-y

**Published:** 2026-09-01

**Authors:** Noura El-Ahmady El-Naggar, Sara M. El-Ewasy, Nancy M. El-Shweihy, Manar G. Helal, Eman M. Sarhan

**Affiliations:** 1 Department of Bioprocess Development, Genetic Engineering and Biotechnology Research Institute, City of Scientific Research and Technological Applications, New Borg El-Arab City, Alexandria 21934 Egypt; 2 Department of Pharmacology and Toxicology, Faculty of Pharmacy, Mansoura University, Mansoura, 35516 Egypt; 3 Biochemistry Department, Faculty of Science, Alexandria University, Alexandria , Egypt

**Keywords:** *Streptomyces glaucescens*, Eumelanin nanoparticles (EuM-NPs), Anti-inflammatory, Molecular docking, COX-1, COX-2, Biochemistry, Biotechnology, Cancer, Chemical biology, Chemistry, Drug discovery, Microbiology

## Abstract

Microbial eumelanin nanoparticles (EuM-NPs) have attracted increasing attention as biocompatible and biodegradable nanomaterials with promising biomedical applications. In the present study, the production of EuM-NPs by *Streptomyces glaucescens* NEAE-H was optimized in a 7-L stirred-tank bioreactor under controlled fermentation conditions. The study investigated the physicochemical characteristics and the in vivo, in vitro, and in silico anticancer and anti-inflammatory activities of EuM-NPs. The maximum EuM-NPs yield reached 367.51 μg/mL after 22 h of cultivation at an agitation rate of 200 rpm. Physicochemical characterization revealed the formation of nearly spherical nanoparticles with particle sizes ranging from 1.23 to 26 nm and a mean diameter of 15.64 ± 4.576 nm. ζ-potential measurements demonstrated good colloidal stability with a surface charge of − 32.6 ± 4.65 mV. Thermogravimetric analysis confirmed high thermal stability, whereas X-ray diffraction analysis verified the characteristic amorphous structure of the produced EuM-NPs. LC–MS/MS analysis detected the oxidative degradation products pyrrole-2,3-dicarboxylic acid (PDCA) [*m/z* 156.9] and pyrrole-2,3,5-tricarboxylic acid (PTCA) [*m/z* 199.9], supporting the presence of eumelanin-derived structural components. In vivo investigations demonstrated significant anticancer activity against Ehrlich ascites carcinoma and solid tumors, reducing tumor volume by up to 78% and tumor weight by up to 83%. Furthermore, EuM-NPs exhibited potent anti-inflammatory activity, achieving 62.62% inhibition of carrageenan-induced paw edema. In vitro cyclooxygenase inhibition assays revealed selective inhibition of COX-2 (IC_50_ 5.61µg/mL , selectivity ratio > 8.91) compared with COX-1 (IC_50_ > 50 µg/mL ) In silico drug-likeness, pharmacokinetics and molecular docking analyses of seven potential eumelanin 5,6-dihydroxyindole (DHI)-precursors highlighted 2,4′-DHI-dimer as the most promising, showing binding affinities of − 5.93 kcal/mol (COX-1) and − 5.27 kcal/mol (COX-2). These findings demonstrate that controlled bioreactor cultivation significantly reduced the production time required to obtain EuM-NPs by *Streptomyces glaucescens* NEAE-H while maintaining comparable production levels and highlighted their potential as multifunctional nanomaterials for anticancer therapy, inflammation management, and future nanomedicine applications.

**Supplementary Information:**

The online version contains supplementary material available at https://doi.org/10.1038/s41598-026-65150-y.

## Introduction

Melanins are heterogeneous biopolymers formed through the oxidative polymerization of phenolic or indolic compounds. They are generally amorphous, high-molecular-weight pigments that typically range in color from brown to black. Owing to their complex polymeric structure, melanins possess unique physicochemical properties, including a negatively charged hydrophobic nature, remarkable resistance to concentrated acids, light, and reducing agents, as well as high thermal stability. Notably, certain melanins can resist thermal decomposition at temperatures up to 600 °C^1,2^. Based on their chemical structures, melanin pigments are generally classified into three distinct types: eumelanin, pheomelanin, and allomelanin^3^. Among these, eumelanin is the predominant form produced in humans and microbes, particularly bacteria and fungi. Eumelanin biosynthesis originates from the amino acid L-tyrosine through a series of enzymatic and nonenzymatic reactions initiated by the enzyme tyrosinase^4^. Initially, L-tyrosine is oxidized by tyrosinase to L-3, 4- dihydroxyphenylalanine (L-DOPA), which is subsequently transformed into dopachrome and finally, melanin is produced by a series of nonenzymatic oxidation–reduction reactions^5^.

Melanins possess a broad range of physicochemical properties, enabling them to function as ultraviolet, X-ray and γ-ray absorbers, amorphous semiconductors, drug carriers, and cation exchangers^6^. Melanins exhibit vital biological activities, including antivenin^7^, antitumor^8^, anti-inflammatory^9^, antimicrobial^10^, and free radicals scavenging activities^10^. Owing to their nanocarrier properties, melanin-based nanomaterials have been extensively explored for biomedical applications, including controlled drug delivery, bioimaging, tissue engineering, bioelectronics, antioxidant therapy, and theranostic interventions^11^. Melanins have also attracted considerable interest owing to their antiviral, radioprotective, and photoprotective properties. They have been reported to protect skin cells against oxidative stress and ultraviolet-induced damage, highlighting their potential use in cosmetic and dermatological formulations. In addition, water-soluble melanins have been incorporated into sunscreens, protective coatings, lenses, paints, and polymeric films to provide protection against ultraviolet radiation^12–14^. Furthermore, microbial melanins exhibit significant radioprotective potential through their capacity to scavenge free radicals and mitigate radiation-induced cellular damage, thereby minimizing the harmful effects of radiation exposure and radiotherapy^15,16^. These properties have also prompted the development of melanin nanoshells as functional radioprotective materials for radiation-shielding applications, including protective coatings, packaging systems, and other technologies intended for high-radiation environments^17^.

Melanin-like nanoparticles (MNPs) have emerged as multifunctional nanomaterials owing to their excellent biocompatibility, biodegradability, antioxidant activity, metal-chelating capacity, photothermal conversion efficiency, and ease of surface functionalization. These unique properties have promoted their application in drug delivery, antioxidative therapy, photothermal therapy, and multimodal imaging. The biological performance of MNPs is strongly influenced by particle size, which affects their biodistribution, circulation time, cellular uptake, and therapeutic efficacy. Ultrasmall nanoparticles (< 20 nm) are particularly advantageous for renal-targeted delivery and rapid clearance. Spherical MNPs (50 to 200 nm) reveal broad utility in ischemic neuroprotection, intracellular reactive oxygen species (ROS) scavenging, and antioxidative food packaging applications. Functionalized MNPs, including gold-hybridized melanin and polydopamine nanostructures ranging from 43 to 190 nm, have exhibited potent antitumor activity against various cancers, including breast and lung carcinomas.

MNPs have demonstrated considerable potential as nanocarriers for drug delivery. For instance, curcumin-loaded melanin nanoparticles have shown therapeutic efficacy against renal fibrosis, while other functionalized MNPs have exhibited beneficial effects in acute kidney injury models^18^. In addition, the strong near-infrared (NIR) absorption and high photothermal conversion efficiency of MNPs enable localized hyperthermia and selective tumor ablation, supporting their application in photothermal cancer therapy with minimal damage to surrounding healthy tissues. Beyond therapeutic applications, MNP-based nanoprobes have been investigated as multifunctional imaging agents for magnetic resonance imaging (MRI), positron emission tomography (PET), and photoacoustic imaging (PAI), highlighting their potential as multifunctional nanomaterials for therapeutic applications of diseases such as prostate cancer^18^.

Microbial melanin production has gained attention in recent years as a greener and more economical alternative to chemical manufacturing^19^. Few microorganisms are capable of producing soluble extracellular melanins^20^. However, the production rate of such melanin is quite low. Soluble, microbial melanin has been produced using recombinant *Escherichia coli*^21^*.* However, the use of recombinant organisms has restrictions based on regulations. Among naturally occurring melanin producers, actinomycetes represent a particularly promising group owing to their ability to synthesize and secrete extracellular dark soluble melanins with potential biotechnological and biomedical value.

In our previous study, *Streptomyces glaucescens* NEAE-H was investigated as a promising producer of extracellular eumelanin under shake-flask cultivation conditions^22^. Production parameters were statistically optimized using Plackett–Burman and face-centered central composite designs, resulting in a maximum eumelanin yield of 350 mg/L after 6 days of incubation. The purified pigment was characterized using UV–visible spectroscopy, Fourier-transform infrared spectroscopy (FTIR), scanning electron microscopy (SEM), and nuclear magnetic resonance (NMR), confirming the characteristic physicochemical features of natural eumelanin. Furthermore, the produced pigment exhibited considerable antioxidant, anticancer, and anti-hemolytic activities, highlighting its potential biomedical relevance.

Microbial EuM-NPs production under controlled bioreactor conditions remains insufficiently explored. Therefore, the present study aimed to investigate the bioreactor-based production of EuM-NPs under controlled fermentation conditions by *Streptomyces glaucescens* NEAE-H in a 7-L stirred-tank bioreactor. The produced EuM-NPs were comprehensively characterized using complementary physicochemical, structural, thermal, and compositional analyses, including TEM, DLS, ζ-potential, EDX, XRD, TGA, and LC–MS/MS. In addition, their biological activities were evaluated through in vivo anticancer and anti-inflammatory studies, supported by in vitro cyclooxygenase inhibition assays and in silico molecular docking and pharmacokinetic analyses. This integrated study combines bioreactor-based production of microbial EuM-NPs with comprehensive physicochemical characterization and multi-level biological evaluation, providing valuable insights into their physicochemical properties, biological activities, and potential biomedical applications.

## Materials and methods

### Materials

Carrageenan λ was purchased from Sigma Aldrich Chemical Co., Ltd. (St. Louis, MO., USA); Diclofenac sodium (Declophen) was gained from PHARCO Pharmaceutical Co., Ltd. (Alexandria, Egypt); Doxorubicin (Dox) (Adricin) was attained from EIMC Pharmaceuticals, (Cairo, Egypt). All other solvents and chemicals used in the current investigational procedures were of analytical or HPLC grade.

### Microorganisms and cultural conditions

In our previous study, *Streptomyces glaucescens* NEAE-H was isolated and identified as an extracellular eumelanin-producing actinomycete^22^. The strain was maintained on starch nitrate agar medium and stored as spore suspensions in 20% (v/v) glycerol at − 20 °C until use in the present study.

### Inoculum preparation

Fifty milliliters of peptone yeast extract iron broth fermentation medium were dispensed in 250 mL Erlenmeyer conical flasks and were inoculated with three 9-mm agar plugs obtained from a 7-day-old stock culture of *Streptomyces glaucescens* NEAE-H. The medium contained the following components (g/L): yeast extract 1; sodium thiosulfate 0.08; ferric ammonium citrate 0.5; K_2_HPO_4_ 1; protease peptone 5; peptone 15, prepared in distilled water and adjusted to pH 7. The inoculated flasks were incubated at 30 °C for 48 h on a rotary shaker incubator at 150 rpm. Subsequently, 5% (v/v) of the previously prepared inoculum was transferred into 250 mL Erlenmeyer shake flask containing 100 mL of fermentation medium and incubated under the same conditions for an additional 48 h.

### EuM-NPs production using a 7-L stirred tank bioreactor under submerged fermentation

Production of EuM-NPs pigment was performed in a stirred tank bioreactor (Cleaver Scientific Ltd.) under submerged fermentation. The total vessel volume was 7 L, while the actual working (culture) volume used in all experiments was 4 L. The bioreactor was equipped with a digitally controlled pH electrode, temperature probe, polarographic DO electrode, and two six-blade Rushton turbine impellers (5.2 cm diameter)”, fixed on the agitator shaft above 3.2 cm air sparger. The pH electrode was calibrated by using standard buffers (Fluka) at pH 7 and 9 prior to the sterilization of the bioreactor (121 °C for 20 min). However, the calibration of the DO electrode was conducted after sterilization by the air until 100% saturation was achieved. The foam was manually controlled by adding a few drops of silicon-based antifoam (Sigma) at foam time. For EuM-NPs production by *Streptomyces glaucescens* strain NEAE-H, fermentation was carried out in RSM optimized medium^22^ of the following composition: L-tyrosine 3; yeast extract 1; sodium thiosulfate 0.08; ferric ammonium citrate 0.5; K_2_HPO_4_ 1; protease peptone 5; peptone 15 and distilled water 1L. The production of EuM-NPs was studied while maintaining the culture’s pH at 7.0 with 2 M sodium hydroxide or 2 M hydrochloric acid throughout fermentation. The stirred tank bioreactor fermentation media were inoculated with 10% (v/v) of the previously prepared inoculum. The temperature of the bioreactor vessel was maintained at 30 degrees Celsius. For the comparison of different stirring speeds’ effect on the EuM-NPs production with pH control, the airflow rate was set to 0.5 vvm using filtered sterile air, and the stirring speed was varied by adjusting the bioreactor’s stirring speed rate to 100, 200, or 300 rpm. After the incubation period, the mycelium was collected by centrifuging the fermentation broth for 15 min at 5000 × *g* to remove cells and debris. The production of EuM-NPs was determined using the cell-free supernatant by measuring optical density (OD) at 250 nm^22^. Based on the obtained results, the optimal stirring speed was determined.

### Cell dry weight determination

During the fermentation process, 10 mL of fermentation broth samples were taken at regular intervals and centrifuged at 5000 × *g* for 15 min in pre-weighed, sterile falcon tubes. Cell dry weight was determined by drying pellets containing cell debris to a constant weight at 50 °C. Whereas the cell-free supernatant was used to determine the EuM-NPs production.

### EuM-NPs purification

EuM-NPs were purified following the method described by El-Naggar & El-Ewasy^22^. Briefly, the supernatant was acidified to pH 2 using 6 M HCl to facilitate nanoparticles precipitation and then allowed to stand for 4 h. The resulting precipitate was collected by centrifugation at 9000 × g for 15 min. The obtained pellets were subsequently washed four times with distilled water, with each washing step followed by centrifugation at 9000 × g for 15 min, to remove residual impurities. Finally, the purified EuM-NPs were lyophilized and stored for further analysis.

### Kinetic calculations

Kinetic parameters including growth rate, productivity, yield coefficients, and dissolved oxygen behavior were analyzed to evaluate process efficiency and metabolic performance.

The specific growth rate (μ) was determined from the slope of the linear regression of the natural logarithm of biomass concentration against cultivation time during the exponential growth phase, according to the relationship μ = (ln X_2_ − ln X₁)/(t_2_ − t_1_).

where X_1_ and X_2_ are the biomass concentrations at times t_1_ and t_2_, respectively.

Volumetric productivity (Q_p_) was calculated as:

$${\mathrm{Q}}_{{\mathrm{p}}} \left( {\mu {\mathrm{g}}/{\mathrm{mL}}/{\mathrm{h}}} \right)\, = \,\left( {{\mathrm{P}}_{\max } \, - \,{\mathrm{P}}_{{0}} } \right)/{\mathrm{t}},$$

where P_max_ is the maximum EuM-NPs production achieved (μg/mL), P_0_ is the initial EuM-NPs production (μg/mL), and t is the cultivation time required to reach the maximum EuM-NPs production, which was 22 h.

The product yield coefficient (Y_p/x_) was determined using biomass and EuM-NPs production at the time of maximum product formation (22 h). The yield coefficient was calculated according to the following equation: Y_p/x_ (μg/g biomass) = P_max_/X_max_.

where P_max_ is the maximum EuM-NPs production (μg/mL), and X_max_ is the biomass concentration (g/L) at the time of maximum EuM-NPs production (22 h).

### EuM-NPs physicochemical characterization

#### Solubility

The solubility of the purified EuM-NPs was determined according to the method developed by Wang et al.^23^ by adding 10 mg of the purified EuM-NPs powder to 100 mL of water, aqueous acids (1N HCl), 1N dilute alkali solutions (such as NaOH, KOH, NH_4_OH, Na_2_CO_3_), and common organic solvents (acetone, hexane, petroleum ether, acetic acid, methanol, ethanol, chloroform, benzene, xylene, ethyl acetate, acetonitrile, dimethylsulfoxide, benzene, isopropyl ether, etc.), stirred for 1 h at 25 °C and filtered. The absorbance of each solution was recorded spectrophotometrically using a UV–visible spectrophotometer at 250 nm to measure the solubility of EuM-NPs^24^.

#### Stability

The thermo-stability, pH stability, and light stability of EuM-NPs were assessed. The thermal stability of the EuM-NPs’ suspended solution was measured after treatment with various temperatures in a thermostatically controlled water bath at 20, 40, 60, 80, and 100 °C for 2,4 and 6 h. Following treatments, the solutions’ absorption was recorded at 250 nm. The light stability of the EuM-NPs’ suspended solution was determined by keeping a 5 mg/mL EuM-NPs solution under the light and the dark for two days. The maximum absorption of the EuM-NPs solution was measured every 6 h intervals at 250 nm. The pH stability of the EuM-NPs pigment was determined by adjusting a 5 mg/mL EuM-NPs solution into a range of pH values (3, 4, 6, 7, 9, 10, and 12) with 0.5 N HCl and 0.5 N NaOH. The EuM-NPs solutions were maintained at 25 °C for 30 min and the maximum absorbance of the centrifuged EuM-NPs solutions was measured at 250 nm.

#### Transmission electron microscope (TEM)

The shape, particle size, EDX (Energy Dispersive X-ray), and mapping analyses of the EuM-NPs were investigated using a carbon-coated copper grid for TEM (JEM-2100 Plus, JEOL Ltd., Japan) at the Central Lab, SRTA-City, Alexandria, Egypt. The size distribution of the synthesized nanoparticles was determined using transmission electron microscopy (TEM). TEM micrographs were analyzed using image analysis software, and the diameters of individual 116 EuM-NPs were measured manually. A histogram was constructed to visualize the particle size distribution, and the mean particle diameter, standard deviation (SD), and particle count (N) were calculated.

#### ζ-potential and particle size analysis

The ζ-potential of the synthesized EuM-NPs was measured using a Malvern Zetasizer instrument. Approximately 1 mL of the purified EuM-NPs suspension was diluted to an appropriate concentration in deionized water to ensure optimal scattering intensity. Measurements were performed at 25 °C. The sample was analyzed in triplicate, and the reported zeta potential values represent the mean of at least three independent measurements. Results are expressed as the mean ± standard deviation. The hydrodynamic diameter and particle size distribution of EuM-NPs were determined using dynamic light scattering (DLS) analysis at 25 °C. The same Malvern Zetasizer instrument was employed for size measurements. The purified EuM-NPs suspension was diluted appropriately to achieve a count rate of 148.6 kcps. The measurements were performed using automatic attenuation settings, and the instrument selected an attenuation level of 10 to optimize the detected scattering intensity during analysis. The measurement duration was 40 s, during which the scattered light intensity data were collected and analyzed to determine the particle size distribution. The measurement position was automatically optimized by the instrument and set at 5.50 mm within the sample cell to obtain an appropriate scattering signal. The Z-average diameter (d.nm) was calculated from the measured data. The polydispersity index (PDI) was determined to assess the uniformity of the particle size distribution.

#### Thermogravimetric analysis (TGA)

Thermogravimetric analysis was performed using a TGA-50H analyzer. The initial sample dried mass of EuM-NPs was analyzed under a dynamic nitrogen atmosphere at a constant flow rate of 40 mL/min. The temperature program employed a linear heating rate of 10 °C/min from ambient temperature (32.5 °C) to 800 °C. Mass loss data were recorded continuously throughout the analysis with sampling intervals of 1 s, providing high temporal resolution of decomposition. Thermal decomposition profiles were analyzed to determine decomposition temperatures and mass-loss patterns.

#### X-ray diffraction (XRD) analysis

The crystallographic characteristics of the produced EuM-NPs were investigated using an X-ray diffractometer (Rigaku, Japan) equipped with Cu Kα radiation (λ = 1.5406 Å). Measurements were performed under the following operating conditions: tube voltage of 40 kV, current of 50 mA, and scanning over a 2*θ* range of 5–80°. Data were collected in continuous scan mode with a step size of 0.01° and a scanning speed of 50° min⁻1.

### LC–MS/MS analysis

Liquid chromatography-mass spectrometry (LC–MS) is a potent analytical method used to separate, identify, and quantify compounds as well as to clarify the structure and chemical characteristics of various molecules. The EuM-NPs sample was characterized by a liquid chromatography instrument that was coupled to a mass spectrometer (Quadrupole LC/MS/MS Mass Spectrometer) with an ionization source for electrospray (AB Sciex Instruments, Triple Quad 5500, Model: 1033104-AH, Serial Number: BB24221205). The analysis was carried out in HPLC column: Agilent Eclipse XDB-C18, 3.5 μM, 4.6 × 100 mm (USA). The temperature in the column oven was set to 30 degrees Celsius. The LC analysis was carried out with a mobile phase consisting of these two solutions: methanol of LC–MS grade mixed with 0.1 percent ammonium hydroxide and MilliQ water mixed with 0.1 percent ammonium hydroxide as the mobile phase. The LC/MS/MS was operated at a flow rate of mobile phase of 0.4 mL/min using a linear gradient of 0.1% ammonium hydroxide and MilliQ water with 0.1% ammonium hydroxide as the mobile phase. The gradient program started with Frac A (95%) and 5% Frac B at 0 min. In each analysis, the injected volume was 60 µL and the total analysis time was 35.43 min. The equilibration time was 5 min. The LC–MS analysis was performed in positive-ion mode using a turbo spray electrospray ionization source. LC–MS were optimized as follows: temperature of ion source (TEM) = 650 °C, Curtain gas (CUR) = 25 psi. Ion source gas 1 (GS1) and ion source gas 2 (GS2) were 50 psi, entrance potential (EP) = − 12 V, turbo ion spray voltage (IS) = − 4.5 kV, declustering potential (DP) = − 26 V.

### Animal ethics

Adult Albino Wistar male rats (n = 18, weight = 250 − 450 g) were acquired from VACSERA, Giza, Egypt. Adult Swiss female albino mice (weight = 20–25 g) were purchased from “Urology and Nephrology Center”, Mansoura, Egypt. Prior to experimentation, all animals were adapted to laboratory circumstances for one week. They also were accommodated under controlled environmental conditions and fed on a normal standardized diet with free access to water and food. All animal experiments were performed in strict accordance with the relevant national and institutional guidelines and regulations governing the care and use of laboratory animals. The study was approved by the Mansoura University Animal Care and Use Committee (MU-ACUC), under approval number MU-ACUC (PHARM.R.23.07.25). All procedures complied with the ARRIVE guidelines.

### In vivo antitumor activity using the Ehrlich solid tumor model

To initiate the Ehrlich solid tumor, animals were inoculated subcutaneously at the right thigh with 200 µL of EAC cell suspension (5 × 10^5^ viable cells/mL) as previously described by Elsherbiny et al.^25^ and El-Naggar et al.^26^. Adult female Swiss albino mice (body weight: 20–25 g) were inoculated subcutaneously into the right thigh with 200 µL of Ehrlich Ascites Carcinoma (EAC) cell suspension at a concentration of 5 × 10^5^ viable cells/mL. The solid tumors were confirmed to develop within 5 days of inoculation (day zero). Animals were then randomly allocated into five groups (n = 8/group) as follows: Group I (EAC Control), in which EAC-bearing mice received normal saline for 21 days and served as the positive control group; Group II (EAC/Dox), in which EAC-bearing mice were treated intraperitoneally with doxorubicin (Dox) at a dose of 2 mg/kg/day for 21 days and served as the standard treatment group; Group III (EAC/ EuM-NPs 5), in which EAC-bearing mice received EuM-NPs at a dose of 5 mg/kg/day for 21 days; Group IV (EAC/ EuM-NPs 10), in which EAC-bearing mice received EuM-NPs at a dose of 10 mg/kg/day for 21 days; and Group V (EAC/ EuM-NPs 20), in which EAC-bearing mice received EuM-NPs at a dose of 20 mg/kg/day for 21 days. Tumor volumes were assessed using a digital caliper in two dimensions every 5 days, with measurements recorded on days 0, 5, 10, 15, and 20^27^.

To manage pain associated with tumor development, meloxicam (5 mg/kg, subcutaneously; Adwic–El Nasr Pharmaceutical Co.) was administered daily from the day of tumor induction and continued as needed based on veterinary assessment. On day 21, 5 mice per group were anesthetized by intraperitoneal injection of thiopental sodium (40 mg/kg; Pharmadrug Production GmbH) and subsequently euthanized via cervical dislocation, blood was collected for serum preparation, and tumor masses were excised, weighed, and preserved in buffered formalin solution for subsequent histopathological examination and immunohistochemical staining. Whereas, three mice per group were maintained for survival analysis. All procedures, including anesthesia and euthanasia, were performed in accordance with relevant international guidelines and regulations, and were approved by the Institutional Animal Care and Use Committee (IACUC).

#### Mean survival time (MST) and percentage increase in life span (% ILS)

Mean survival time (MST) and percentage increase in life span (%ILS) were estimated according to the following formulas^25^: MST = ∑ [survival time (days) of each mouse in a group]/(total number of mice) and %ILS = [(MST of the treated group)/(MST of the control group)-1] × 100.

#### Tumor’s volume and weight determination

The tumor volume was calculated using the formula A x B^2^ × 0.5, where “A” denotes the diameter of the largest diameter of the tumor and “B” denotes its perpendicular. At the end of the experimentations, the weight of each separate tumor mass was documented.

#### Lipid peroxide (LPO) and total antioxidant capacity (TAC) evaluation

Serum levels of lipid peroxides, principally malondialdehyde (MDA), and total antioxidant capacity (TAC) were detected using commercially available colorimetric Biodiagnostics assay kits (Biodiagnostics Co, Giza, Egypt) according to the producer’s directions.

#### Histopathological examination of the tumor masses

The fixed tumor masses were then embedded in paraffin and partitioned into 5 μm slices. Hematoxylin and Eosin (H&E)-stained portions were observed by a blind pathologist. Immunohistochemical revealing of caspase-3 was done using 5-μm tumor slices by standard immunohistochemical staining. The specimens were deparaffined with xylene. Briefly, the mouse primary monoclonal active caspase-3 antibody (Millipore, AB3623) was diluted in a ratio of 1:100, and incubated with the specimens overnight at 4◦ C in a humidified chamber. Then, the slides were washed with phosphate buffer saline (PBS) and a second biotin-labeled rabbit anti-mouse IgG (Dako code E035401) which worked as a secondary antibody.

### In vivo antitumor activity using Ehrlich ascites carcinoma model

To initiate the Ehrlich ascites carcinoma, mice were divided randomly into the following experimental groups, with eight animals per group: Group I, normal control, received normal saline without EAC-cell inoculation; Group II, EAC control, was intraperitoneally inoculated with EAC cells (2 × 106 cells/mouse) and received normal saline; Group III, EAC/5-FU, was inoculated with EAC cells and treated with 5-fluorouracil (5-FU; 10 mg/kg/day) as the standard treatment^28^; and Group IV, EAC/EuM-NPs, was inoculated with EAC cells and treated with EuM-NPs at 5 mg/kg/day. Except for the normal control group, all mice were inoculated intraperitoneally with 2 × 106 EAC cells per mouse. Treatment with 5-FU or EuM-NPs was initiated 24 h after tumor-cell inoculation and continued once daily for 10 consecutive days^28^. At 24 h after the final treatment, following an 18-h fasting period, five mice from each group were weighed and sacrificed. Blood samples were harvested for the evaluation of hemoglobin (Hb) content and counts for red (RBC) and white blood cells (WBC). Ascitic fluid was collected from the peritoneal cavity to determine tumor-cell volume and total tumor-cell count. The count of viable cells and non-viable cells was assessed by centrifugation of the ascetic fluid and staining with trypan blue (0.4% in normal saline) and another portion was centrifuged in a graduate centrifuge tube at 1,000 rpm for five min and the packed cell volume was calculated. The remaining three mice in each group were monitored to calculate the mean survival time (MST) and percentage increase in life span (%ILS). All analytical measurements were performed in triplicate.

### In vivo anti-inflammatory carrageenan-induced rat hind paw edema model

The anti-inflammatory effect of EuM-NPs was estimated using the carrageenan-induced paw edema method in rats following the method described by Khanna et al.^29^. The formerly acclimatized adult albino *Wistar* male rats (n = 18, weight 250 − 450 g) were allocated into three groups (n = 6/group). Then, 0.1 ml of 1% carrageenan suspension, immediately prepared before use, was inoculated in the sub-planter tissue of the right animal’s hind paw to provoke paw inflammation and edema. One hour before carrageenan injection, the rats were pretreated as follows: Group I, Control group, I.P. injected with the vehicle (sterile distilled water) and was used as an untreated control; Group 2, Diclofenac-treated group, injected with diclofenac sodium (10 mg/kg, I.P.) as an anti-inflammatory reference agent^30^; Group 3, EuM-NPs -treated group, injected with EuM-NPs (10 mg/kg, I.P.). EuM-NPs was prepared as a suspension in sterile distilled water. Diclofenac sodium was dissolved in sterile distilled water. The left hind paw remained untreated considering it as a control to evaluate the change in paw weight. Carrageenan triggered evident redness and swelling which was well established after 3 h and continued till the end of the experiment. Four hours following carrageenan injection, rats were sacrificed then the right and the left hind paws of each animal were removed at the tibiotarsic articulation and weighed for calculating the inhibition percentage in paw edema^31^. The difference in weight between the right and left paws was documented for each animal. The percentage increase in weight of the carrageenin injected right hind paw above the left hind paw was estimated and the percentage reduction of edema from the control group was used as an index of the anti-inflammatory effect.

###  COX-1 and COX-2 inhibition assay

The anti-inflammatory effectiveness of EuM-NPs was also assessed in vitro by comparing its inhibitory ability on the COX-1 and COX-2 enzyme to the reference drug celecoxib by an enzyme immune-assay (EIA) technique (Catalog No. 560101, Cayman Chemicals, Ann Arbor, MI) following previous studies^32,33^.

### In silico computational experiments

Molecular docking simulation analysis using Autodock v.4.2 was performed on a Linux-based operating system installed on an Intel(R) Core(TM) i7-4710HQCPU ×64-bit-based processor with 2.50 GHz processing speed, and equipped with 8.00 GB RAM. Drug-protein interaction (DPI) pharmacological network analysis and visualization using Cytoscape v.3.10.2 software was carried out on Sonoma 14.5 MacOS installed on M1 Apple Ship with 8 GB RAM. ChemAxon’s MarvinSketch v23.17.0 software was used to sketch out the compound chemical structures. Schrodinger suite 2021-2 was used for energy minimization, while UCSF-Chimera v.1.17.3 software was used for protein structure visualization and manipulation.

### Compound retrieval and ligand preparation

2D structure of both polycyclic black chromogenic eumelanin [ReaxysID 27112591] and its monomeric building block 5,6-dihydroxy indole (DHI) [PubChem CID 114683; ReaxysID 122055] have been retrieved in structural data format (SDF) from REAXYS (http://www.reaxys.com/, accessed on Jan. 9, 2024) database, while those naturally probable fragments of DHI-based oligomeric precursors including DHI-dimers (2,2′-bi-indolyl [PubChem CID 46173557], 2,4′-bi-indolyl and 2,7′-bi-indolyl), linear trimer and macrocyclic tetramer have been sketched using ChemAxon’s MarvinSketch v23.17.0 software (https://chemaxon.com/). Prior to molecular docking analysis, all the studied ligands were structurally optimized and energy minimized using OLPS4 forcefield with the default maximum of 2500 iteration runs and gradient coverage threshold of 0.05 using the Schrodinger suite (Release 2021-2).

### Drug-likeness and pharmacokinetics (ADME/Tox) analysis

Physicochemical prosperities, pharmacokinetics, and drug-likeness nature of the polycyclic eumelanin and its mono/oligomeric precursors were predicted using SwissADME (http://www.swissadme.ch/; accessed on Jan. 10, 2024)^34^.

### Network pharmacology and target prediction

The possible protein targets predicted for each of the seven tested DHI-based fragments have been retrieved from Swiss Target Prediction (http://www.swisstargetprediction.ch/, accessed on Apr. 10, 2024) server. On the other hand, about 15,391 inflammatory biomarkers were retrieved from GeneCards (https://www.genecards.org, accessed on Apr 10, 2024) database, and were then filtered to get the most significant 250 inflammatory protein targets with the highest ‘relevance score’ values. Intersect of the predicted potential targets of DHI-based fragments from the SwissTargetPrediction server, and those of inflammatory-related protein biomarkers from the GeneCards database, was carried out, and overlapping targets were visualized as a network constructed by Cytoscape v.3.10.2 software.

### Macromolecular targets’ retrieval, preparation, and active site prediction

X-ray crystal structure of the inflammatory macromolecular targets’ cyclooxygenase-1 (COX-1; PDBID: 4O1Z) and cyclooxygenase-2 (COX-2; PDBID: 4PH9) has been selected and retrieved from the ‘Protein Data Bank’ (https://www.rcsb.org, accessed on May 22, 2024) server. Prior to molecular docking both macromolecular targets (4O1Z and 4PH9) have been optimized and energy minimized using OLPS_2005 forcefield using Schrodinger macromolecule preparation wizard (Release 2021-2). 3D geometrical coordinates and active site pocket dimensions have been computed using AutoSite-1.1 from AGFRsuite v.0.1.

### Molecular docking and validation

In silico molecular docking simulation of the whole studied ligands on the active site of both cellular targets [4O1Z and 4PH9] was performed using AutodockTools v.1.5.7. X, Y, and Z grid-box coordinates were assigned for both COX-1 (252.226 X 104.982 X 5.671, with dimensions of 40X48X46) and COX-2 (11.026 X 19.048 X 27.018, with dimensions of 52 X 54 X 40), with the default spacing of 0.375°A. Then Autodock4.2 binaries were used to perform rigid molecular docking with Lamarckian Generic Algorithm (GA) of 250 iteration run. The co-crystalized inhibitors of COX-1 (MXM: Meloxicam) and COX-2 (IBP: Ibuprofen) were re-docked on their co-crystalized macromolecules [4O1Z and 4PH9, respectively] for validating issues. Celecoxib (CEL) was also used as a known standard cyclooxygenase (COX) inhibitor drug. All generated docked conformers were then examined and those with the best lowest binding energy (ΔG) poses were selected for further ligand–protein interaction analysis and visualization using UCSF Chimera v.1.17.3.

### Statistical significance

Statistical significance was evaluated using GraphPad Prism (version 9.0; GraphPad Software, San Diego, CA, USA).

## Results and discussion

In our previous study^22^, *Streptomyces glaucescens* NEAE-H was identified as an efficient producer of extracellular eumelanin. Following statistical optimization under shake-flask conditions, the maximum eumelanin yield reached 350 mg/L. The pigment was recovered by acid precipitation and exhibited a characteristic UV–visible absorption profile, with strong absorption in the ultraviolet region and a maximum absorption peak at 250 nm.

### EuM-NPs production under different stirring speeds and controlled pH conditions

**Fig. 1:**
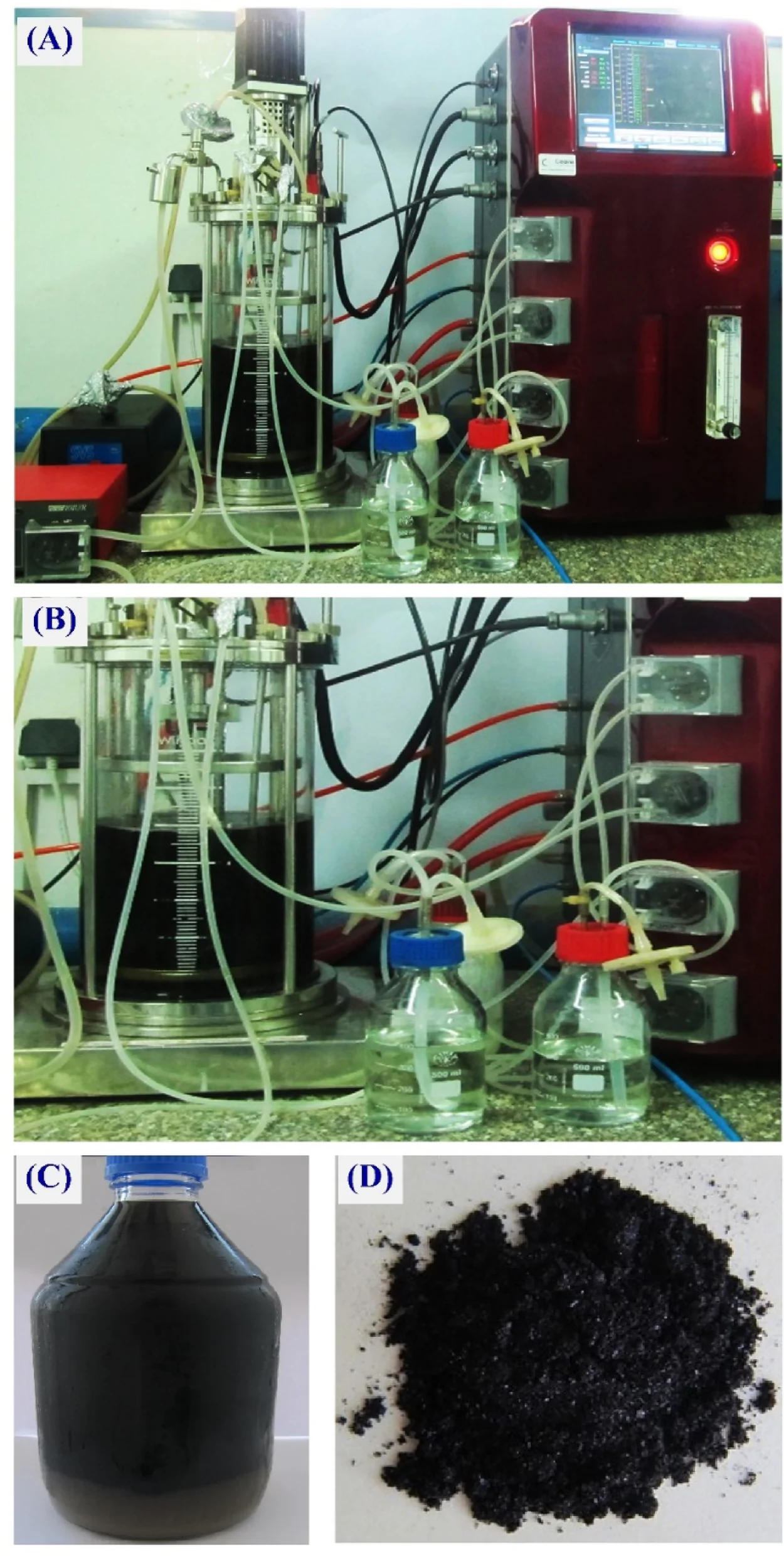
(**A**, **B**) EuM-NPs production in the bioreactor; (**C**) the collected culture with EuM-NPs after fermentation; (**D**) Granules of the extracted lyophilized EuM-NPs.

**Fig. 2:**
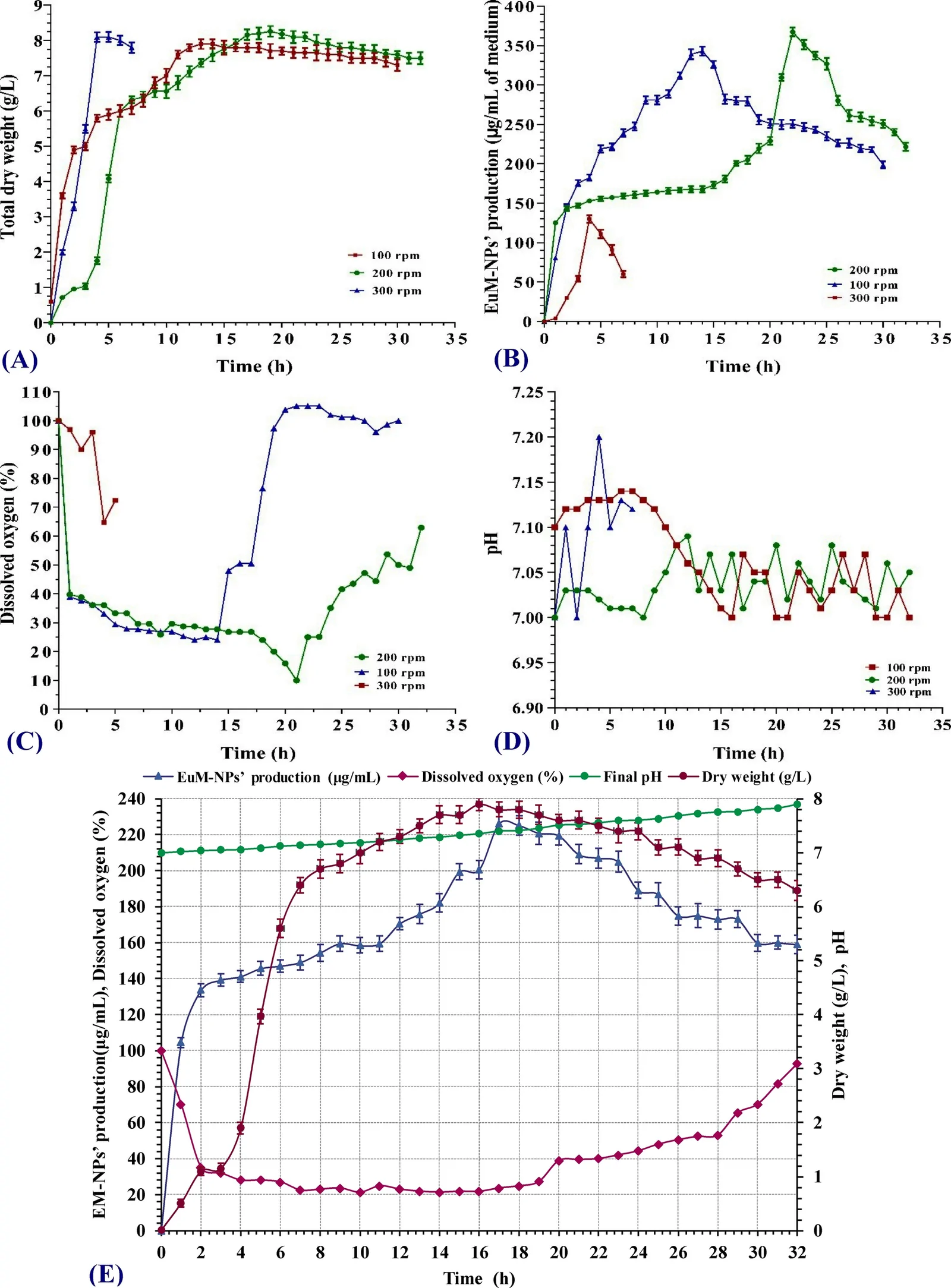
Time-course profile of (**A**) total dry weight; (**B**) EuM-NPs production; (**C**) dissolved oxygen and (**D**) final pH during cultivation of *Streptomyces glaucescens* NEAE-H in 7L stirred tank bioreactor under different stirring speeds and controlled pH. (**E**) Time-course profiles of total dry weight, EuM-NPs production, dissolved oxygen, and final pH under uncontrolled pH conditions.

The effects of different stirring speeds (100, 200, and 300 rpm) on biomass accumulation, dissolved oxygen (DO), pH variation, and EuM-NPs production by *Streptomyces glaucescens* NEAE-H in a 7 L stirred-tank bioreactor operated under controlled pH conditions are illustrated in Figs. 1A–D and 2. Figure 1A, B depicts EuM-NPs production during the fermentation process in the bioreactor. Figure 1C shows the harvested fermentation broth containing EuM-NPs after completion of the cultivation process, whereas Fig. 1D presents the physical appearance of the purified lyophilized EuM-NPs with a true black color obtained following downstream processing. It was suggested that melanin polymers constitute the building blocks of melanin granules^35^.

As shown in Fig. 2A, stirring speed markedly affected biomass accumulation. At 100 rpm, the total dry weight increased rapidly during the early stages of fermentation, reaching approximately 7.9 g/L after 13–14 h, followed by a slight decline toward the end of cultivation. A comparable maximum biomass concentration was obtained at 200 rpm, although growth exhibited a short lag phase during the first few hours before increasing steadily to reach the highest dry cell weight (8.26 g/L) at 19 h. In contrast, cultivation at 300 rpm promoted the fastest initial growth, with biomass reaching about 8.1 g/L within 4–5 h. However, the fermentation was terminated at an earlier stage, suggesting that excessive agitation accelerated growth but did not support prolonged cultivation.

The production profile of EuM-NPs (Fig. 2B) demonstrated that agitation intensity strongly influenced EuM-NPs biosynthesis. At 100 rpm, EuM-NPs production gradually increased throughout cultivation, reaching a maximum of approximately 342.91 μg/mL at 14 h, after which a gradual decline was observed. The highest EuM-NPs yield was achieved at 200 rpm, where production remained relatively stable during the initial growth phase before sharply increasing to 367.51 μg/mL after 22 h of cultivation. Thereafter, production slowly decreased but remained higher than that obtained at the other agitation rates.

Conversely, 300 rpm resulted in considerably lower EuM-NPs productivity, with a maximum concentration of only 130.12 μg/mL observed during the early fermentation stage, followed by a rapid decline. Although increasing agitation generally enhances nutrient distribution and oxygen availability, excessive stirring may exert shear stress capable of damaging filamentous mycelia, disrupting pellet morphology, and consequently reduce secondary metabolite biosynthesis, including EuM-NPs production by shifting cellular resources toward stress adaptation, cellular maintenance and survival. Moderate agitation provides optimal oxygen transfer while preserving the physiological state required for efficient metabolite production. Maximum EuM-NPs production in the shake flask was obtained at an agitation speed of 100 rpm. Further increase of agitation speed to 200 rpm decreased the amount of melanin produced^22^.

The DO profiles further supported these observations (Fig. 2C). At 200 rpm, DO decreased rapidly from near saturation to approximately 20–30%, reflecting active oxygen consumption associated with biomass growth and EuM-NPs synthesis. The DO level subsequently declined to nearly 10% around the time of maximum product formation before increasing again as growth slowed and oxygen demand decreased. At 100 rpm, DO remained relatively low (approximately 25–40%) during most of the cultivation period, indicating oxygen limitation that likely restricted the maximum EuM-NPs yield despite supporting satisfactory biomass formation. In contrast, 300 rpm maintained DO values close to saturation (90–100%) after the early cultivation stage, indicating that oxygen was not limiting under these conditions. Nevertheless, the poor EuM-NPs productivity observed at this agitation rate suggests that excessive shear stress rather than oxygen availability became the dominant factor affecting metabolite biosynthesis.

The pH profiles remained relatively stable throughout the fermentation process (Fig. 2D), fluctuating within a narrow range of approximately 7–7.14 under all stirring conditions. The limited pH variation confirms the effectiveness of the pH control strategy and indicates that the differences observed in growth and EuM-NPs production were primarily attributable to the imposed agitation rates rather than pH-related effects. The findings demonstrate that agitation is a critical operational parameter governing both physiological behavior and EuM-NPs biosynthesis by *Streptomyces glaucescens* NEAE-H. Among the tested conditions, 200 rpm provided the most favorable balance between oxygen transfer and shear stress, resulting in the highest EuM-NPs production, whereas 100 rpm appeared to impose oxygen-transfer limitations and 300 rpm exerted detrimental hydrodynamic effects on metabolite synthesis. Therefore, 200 rpm was identified as the optimal stirring speed for EuM-NPs production in the 7 L stirred-tank bioreactor under controlled pH conditions.

Microbial growth and the synthesis of their metabolites in bioreactors are significantly influenced by medium components and physical parameters, including fermentation time, temperature, aeration, and dissolved oxygen^36^. During the fermentation process, aeration provides the oxygen necessary for cell growth in addition to eliminating exhaust gases^37^. However, agitation can result in mixing and shear forces during the fermentation process. This allows oxygen, heat, and nutrients to be thoroughly mixed and efficiently transferred in the fermentation broth. It also breaks up the air into tiny bubbles, increasing the surface area of contact with the gas and the liquid and preventing mycelia from clumping, which increases their ability to absorb oxygen^37^. The effect of agitation on microorganisms can be seen in several ways, including alterations in morphology, variations in metabolite synthesis and growth, and damage to cell structures^36^. When the speed of the agitation is too slow, the viscosity of the fermentation broth increases, resulting in a decrease in mass transfer efficiency^38^. In contrast, high agitation speed raises power consumption and generates heterogeneous mixing and shear forces that can harm fragile microorganisms and disrupt product production^39^.

To the best of our knowledge, there is a scarcity of studies investigating the kinetic behavior of melanin production by actinomycetes under bioreactor fermentation conditions, as most previous reports have been conducted using shake-flasks. Under flask culture conditions, previous studies have demonstrated considerable variation in melanin production ranging between 0.097^40^ and 21.13 g/L^41^ by different actinomycete strains with respect to incubation time. Moreover, Kordjazi et al.^42^ reviewed several strategies used to improve melanin production by *Streptomyces*, including optimization of cultivation parameters, precursor supplementation, utilization of low-cost substrates, and process engineering approaches. *Streptomyces antibioticus* NRRL B-1701 exhibited relatively rapid melanin biosynthesis, achieving a maximum yield of 0.24 g/L within only 36 h of shake flask cultivation. Likewise, *Streptomyces nigra* produced approximately 2.45 g/L after 72 h of incubation^43^, indicating a comparatively shorter production cycle with higher productivity. In contrast, several strains required longer incubation periods to attain maximum melanin yields. *Actinoalloteichus* sp. MA-32 reached its highest melanin concentration (97 μg/mL) after 120 h of cultivation^40^. Similarly, *Streptomyces glaucescens* supplemented with 5 mM caffeic acid produced 125.25 ± 6.01 mg/L after 144 h^44^, while *Streptomyces glaucescens* NEAE-H achieved 350 mg/L after 6 days (144 h) of incubation^22^. Extracellular pigment production by *Streptomyces parvulus* also required prolonged fermentation, reaching 465.3 μg/mL after 7 days of incubation^45^. The longest incubation period was reported by Vasanthabharathi et al.^41^, where crude melanin production exceeded 21.13 g/L after 168 h of shake flask fermentation. In another study, Restaino et al.^46^ described extracellular melanin production by *Streptomyces nashvillensis* and highlighted that medium composition and fermentation conditions markedly affected pigment productivity. A maximum melanin concentration of 0.74 ± 0.01 g/L was achieved after 96 h of shake flask cultivation^46^. Subsequently, Restaino et al.^47^ demonstrated that supplementation with selected metal ions substantially enhanced melanin biosynthesis in the same strain. A maximum melanin production of 4.0 ± 0.1 g/L was obtained in shake flasks flask culture. Fermentation experiments in stirred-tank bioreactors enabled process operation under controlled conditions and further enhanced pigment production up to 4.9 ± 0.1 g/L^47^. Supplementation of the production medium with 0.04% CuSO_4_·5H_2_O significantly stimulated eumelanin production by *Streptomyces lasalocidi* NTB 42, resulting in 875 mg/g dry weight of eumelanin after 10 days of shake flask cultivation^48^. More recently, Cimini et al.^49^ reported a newly isolated *Streptomyces nigra* strain with improved melanin production potential, which produced approximately 2.45 g/L of melanin after 72 h of incubation in 250 mL shake flasks, whereas the present study achieved 367.51 μg/mL (0.3675 g/L) within only 22 h under controlled bioreactor conditions.These studies indicate that melanin biosynthesis kinetics differ significantly among microbial strains, with some organisms exhibiting rapid pigment production within, whereas others require extended cultivation periods of 120–168 h to achieve maximum yields.

### Biomass growth, EuM-NPs production, and dissolved oxygen under 200 rpm and uncontrolled pH conditions in a stirred-tank bioreactor

The time-course profiles of biomass (dry weight, g/L), EuM-NPs production, dissolved oxygen (DO), and pH variation during the cultivation of *Streptomyces glaucescens* NEAE-H in a 7 L stirred-tank bioreactor under uncontrolled pH conditions are presented in Fig. 2E. As shown in Fig. 2E, the biomass dry weight increased rapidly during the early stages of fermentation. The total dry weight rose from approximately 0.007 g/L at inoculation to 1.90 g/L after 4 h and increased sharply thereafter, reaching 5.60 g/L at 6 h and 6.70 g/L at 8 h. Biomass accumulation continued during the exponential phase, attaining a maximum value of 7.90 g/L after 16 h of cultivation. Subsequently, a gradual decline was observed, with the dry weight decreasing to 6.30 g/L by the end of the fermentation period (32 h), which may be attributed to nutrient depletion and the onset of the stationary and decline phases.

EuM-NPs biosynthesis started immediately after inoculation and exhibited a rapid increase during the early stages of cultivation. The EuM-NPs concentration increased rapidly from 0.04 to 104.52 μg/mL within the first hour and reached 145.70 μg/mL after 5 h. Thereafter, production increased gradually during active growth, reaching 182.15 μg/mL at 14 h and 200.60 μg/mL at 16 h. The highest EuM-NPs yield (226.10 μg/mL) was obtained after 17 h of fermentation. Following this peak, EuM-NPs production gradually declined to 159 μg/mL at 32 h.

The dissolved oxygen profile reflected the metabolic activity of the culture throughout fermentation. The DO concentration decreased sharply from 100% at the beginning of cultivation to 35% within 2 h and further declined to approximately 21–28% between 4 and 19 h, corresponding to the period of rapid biomass accumulation and active EuM-NPs production. After reaching the maximum EuM-NPs concentration, the DO level increased progressively from 38.9% at 20 h to 92.7% at the end of cultivation. This increase in residual oxygen likely resulted from reduced oxygen consumption as cell growth slowed and the culture entered the stationary and decline phases.

Under uncontrolled conditions, the pH of the fermentation broth exhibited a continuous upward trend throughout the cultivation period. The initial pH of 7 increased gradually to 7.19 after 10 h and reached 7.45 at 19 h. Thereafter, the pH continued to rise steadily, reaching 7.89 by the end of fermentation. The alkalization of the culture medium may be associated with the utilization of nitrogenous substrates and the accumulation of alkaline metabolites during the later stages of growth. These findings indicate that EuM-NPs production by *Streptomyces glaucescens* NEAE-H under uncontrolled pH conditions was closely associated with active growth and was maximized during the late exponential phase. However, the gradual increase in medium pH beyond the optimal range after 17 h coincided with a decline in EuM-NPs production despite the availability of dissolved oxygen. This observation suggests that uncontrolled pH conditions may adversely affect the activity of enzymes involved in EuM-NPs biosynthesis and ultimately limit EuM-NPs production. Therefore, maintaining an appropriate pH environment appears to be crucial for maximizing EuM-NPs production during bioreactor cultivation.

### Kinetic analysis of biomass growth and EuM-NPs production in a stirred-tank bioreactor under controlled and uncontrolled pH conditions

The kinetic performance of *Streptomyces glaucescens* NEAE-H under controlled (pH 7) and uncontrolled pH conditions was evaluated in a 7-L stirred-tank bioreactor operated at 200 rpm and 30 °C. Clear differences were observed in both biomass growth and EuM-NPs production, as well as in dissolved oxygen (DO) profiles (Table 1).

**Table 1:** Comparative kinetic and process performance analysis of cell growth and EuM-NPs production by *Streptomyces glaucescens* NEAE-H in a 7-L stirred-tank bioreactor operated at 200 rpm and 30 °C under controlled and uncontrolled pH conditions.

Kinetic parameters	Stirring speed, 200, 30 °C	
	Controlled pH (7)	Uncontrolled pH
*Biomass kinetics*		
Initial biomass (X_0_) (g/L)	0.004	0.007
Maximum biomass (X _max-biomass_) (g/L)	8.26	7.90
Time to X _max-time_ (h)	19	16
Maximum specific growth rate (μ_max_) (h⁻^1^)	0.44	0.43
Maximum biomass formation rate (dX/dt) _max_ (g/L/h)	2.32	2.07
*EuM-NPs production*		
Initial EuM-NPs concentration (P_0_) (μg/mL)	0.02	0.04
Maximum EuM-NPs concentration (P _max-vol_) (μg/mL)	367.51	226.10
Time (t) to P _max-time_ (h)	22	17
Overall volumetric productivity (Q_p_) (μg/mL/h)	16.70	13.30
Product yield coefficient (Y_p/x_) (mg/g biomass)	46.88	28.99
Post-peak melanin decrease (μg/mL)	145.85	67.10
Post-peak loss (%)	39.69	29.68
*Dissolved oxygen*		
Dissolved oxygen at P_max_ (%)	25	23.6
Minimum dissolved oxygen (DO_min_ (%)	10	21.3
Maximum dissolved oxygen (DO_max_) (%)	100	100
DO operating range (%)	90	78.7

μ_max_ was calculated from the slope of ln(X) vs time during exponential growth using linear regression. Volumetric productivity (Q_p_) was calculated as (P_max_ − P_0_)/t. t corresponds to the cultivation time required to reach maximum EuM-NPs concentration. Y_P/X_ was calculated using the biomass and EuM-NPs concentrations at the time of maximum product formation.

For biomass kinetics, both cultures exhibited rapid growth during the initial cultivation period. Under controlled pH conditions, the biomass concentration increased from 0.004 g/L to a maximum of 8.26 g/L after 19 h, whereas the uncontrolled culture reached a slightly lower maximum biomass concentration of 7.90 g/L after 16 h. The maximum specific growth rates were comparable, reaching 0.44 and 0.43 h⁻^1^ under controlled and uncontrolled pH conditions, respectively. Similarly, the maximum biomass formation rate was slightly higher under controlled pH (2.32 g/L/h) than under uncontrolled pH (2.07 g/L/h), indicating that maintenance of pH at neutrality provided a more favorable environment for microbial growth and metabolic activity.

Regarding EuM-NPs production, a pronounced effect of pH control was observed on EuM-NPs biosynthesis. The controlled culture achieved a maximum EuM-NPs concentration of 367.51 μg/mL after 22 h, whereas only 226.10 μg/mL was obtained under uncontrolled pH conditions after 17 h. Therefore, pH regulation resulted in approximately 1.63-fold enhancement in EuM-NPs production. Under controlled pH conditions, EuM-NPs concentration decreased from 367.51 to 221.66 μg/mL by the end of fermentation, corresponding to a post-peak loss of 145.85 μg/mL (39.69%). Under uncontrolled pH conditions, the product concentration declined from 226.10 to 159.00 μg/mL, resulting in a smaller decrease of 67.10 μg/mL (29.68%). Although the relative product loss was lower in the uncontrolled culture, the substantially higher peak concentration achieved under controlled pH more than compensated for this decline, yielding significantly greater final production and process performance.

The higher productivity under controlled pH was further confirmed by the overall volumetric productivity (Qp), which increased from 13.30 μg/mL/h under uncontrolled pH to 16.70 μg/mL/h under controlled pH. In addition, the product yield coefficient (Y_P/X_), further confirmed the superiority of controlled pH conditions, increased substantially from 28.99 to 46.88 μg/g biomass, demonstrating that pH control improved not only biomass formation but also provided superior overall process performance and achieved the highest EuM-NPs yield, highlighting the importance of pH regulation as a key operational parameter for large-scale EuM-NPs production. Melanin biosynthesis in *Streptomyces* species is catalyzed by tyrosinase and related phenol oxidases, which are highly sensitive to pH. The maintenance of neutral pH 7 in controlled pH likely provided an optimal condition for these enzymes required for melanin biosynthesis, allowing an extended fermentation period, resulting in higher EuM-NPs accumulation. In contrast, the progressive pH increases in uncontrolled pH (from 7.0 to 7.89) may have shifted the pH away from the enzyme optimum, contributing to declining melanin production rates.

The dissolved oxygen (DO) profile revealed a strong association between oxygen consumption and microbial growth. In both cultures, DO decreased rapidly during the exponential growth phase due to increased respiratory activity. Under controlled pH conditions, EuM-NPs production reached its maximum at a DO level of 25%, while the minimum DO value recorded during cultivation was 24%. In contrast, maximum EuM-NPs production under uncontrolled pH occurred at a slightly lower DO value (23.6%), with a minimum DO of 21.3%. These findings suggest that EuM-NPs biosynthesis is favored under moderate oxygen-limited conditions.

The kinetic analysis clearly demonstrates that controlled pH operation is superior to uncontrolled cultivation for EuM-NPs production by *Streptomyces glaucescens* NEAE-H. The controlled process provided higher biomass concentration, greater volumetric productivity, improved product yield, and about 62.5% increase in maximum EuM-NPs concentration, highlighting pH regulation as a critical operational parameter for large-scale production of EuM-NPs.

### EuM-NPs’ physicochemical characterization

#### Solubility profile of EuM-NPs

The solubility of EuM-NPs produced by *Streptomyces glaucescens* was tested across a broad range of solvents. The purified EuM-NPs exhibited a characteristic solubility pattern (Supplementary Table 1). The pigment was soluble in water, alkaline solutions (1N NaOH, KOH, Na_2_CO_3_ solution, and NH₄OH), methanol, and DMSO. In contrast, it was insoluble in ethanol, acetone, chloroform, ethyl acetate, benzene, xylene, hexane, acetonitrile, acetic acid, petroleum ether, and 1N HCl.

The pigment exhibited high solubility in alkaline media, which can be attributed to the ionization of phenolic and carboxylic functional groups present within the melanin polymer. Deprotonation of these groups under basic conditions increases the net negative charge and enhances intermolecular repulsion, resulting in improved dissolution. The insolubility of the pigment in most organic solvents and acidic conditions reflects its highly cross-linked, hydrophobic aromatic structure and extensive intermolecular interactions. Acidification promotes protonation of functional groups, leading to aggregation and precipitation of melanin particles.

#### Thermal stability

The thermal stability of EuM-NPs produced by *Streptomyces glaucescens* was evaluated by measuring absorbance at 250 nm over a temperature range of 20 to 100°C at three exposure durations: 2, 4, and 6 h (Supplementary Fig. 1A). The results indicated that EuM-NPs exhibited high thermal stability, particularly at shorter exposure durations. After 2 h of incubation, absorbance values remained nearly constant across the tested temperature range, decreasing only slightly from 0.873 at 20 °C to 0.867 at 100 °C, indicating minimal impact on structural integrity under short-term heat exposure. However, a time-dependent decrease in stability was observed with prolonged heating. At 4 h, a gradual reduction in absorbance was recorded from 0.873 to 0.855, while at 6 h the decrease was more pronounced, reaching 0.837 at 100 °C. This trend suggests that extended thermal exposure may induce partial structural modifications or gradual degradation of the melanin polymer.

Despite this slight decline, the overall reduction in absorbance remained limited, confirming the strong thermal stability of the pigment. These findings indicate that EuM-NPs produced by *Streptomyces glaucescens* possesses suitable stability for applications involving thermal processing or sterilization.

#### Stability under light and dark conditions

The photostability of EuM-NPs was evaluated by measuring absorbance at 250 nm over 48 h under continuous light exposure and dark storage conditions (Supplementary Fig. 1B). The results showed a clear distinction between the two conditions. Under dark conditions, absorbance remained unchanged throughout the experiment (0.875), indicating high stability and the absence of degradation, hydrolysis, or oxidation in the dark. In contrast, sample exposed to continuous light exhibited a gradual decrease in absorbance over time, declining from 0.875 at 0 h to 0.863 after 48 h (about 1.4% reduction). The overall change was minimal, indicating strong inherent photostability of the EuM-NPs produced by *Streptomyces glaucescens*. These findings support its suitability for applications involving light exposure, such as cosmetic, sunscreen, and UV-protective materials, while also suggesting that storage under dark conditions may further enhance long-term stability.

#### pH stability

The effect of pH on EuM-NPs stability was evaluated over a wide range (pH 3–12) by monitoring absorbance at 250 nm (Supplementary Fig. 1C). The results revealed a clear pH-dependent behavior. At pH 3, a significant decrease in absorbance (0.440) was observed, indicating pronounced instability under strongly acidic conditions. This reduction is attributed to protonation of surface functional groups (e.g., carboxyl and phenolic moieties), which decreases electrostatic repulsion and promotes nanoparticles aggregation, thereby reducing the optical response. From pH 4 onward, absorbance increased markedly (0.776–0.78 at pH 4–6). Maximum and stable absorbance values (0.837–0.847) were recorded across pH 7–12, indicating excellent stability under neutral and alkaline conditions. The negligible variation within this range suggests that EuM-NPs maintains its structural integrity without evidence of alkaline degradation even at pH 12.

#### Transmission electron microscope (TEM)

**Fig. 3:**
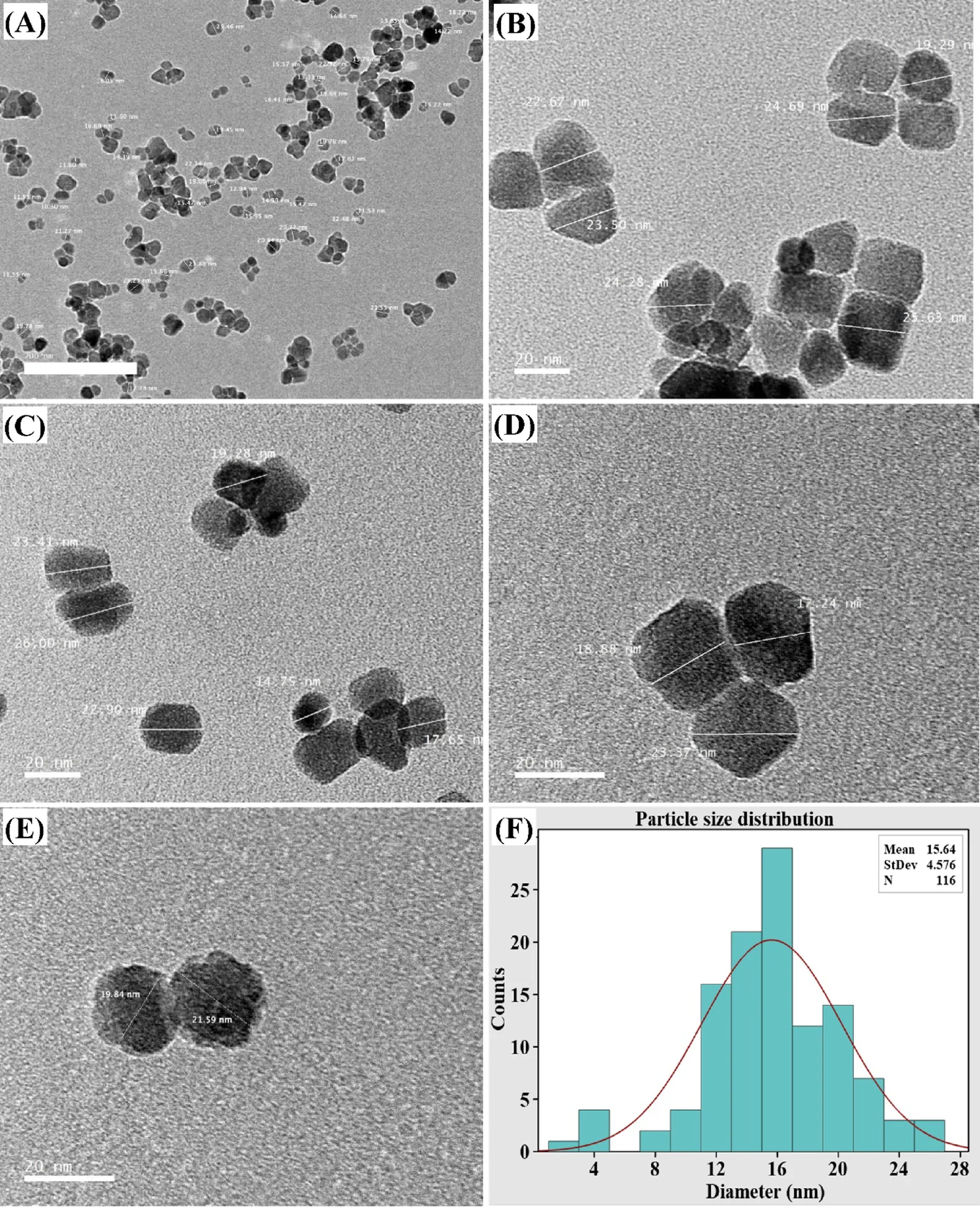
(**A**–**E**) Transmission electron microscopy micrographs of the extracted EuM-NPs granules captured at different magnifications, illustrating their morphology, and dimensions. (**F**) Particle-size distribution histogram of 116 measured nanoparticles.

TEM analysis was carried out to characterize the shape and particle size of the EuM-NPs. Figure 3 depicts the TEM micrographs of the extracted EuM-NPs’ granules. The TEM images (Fig. 3A-E) show nearly spherical nanoparticles of EuM-NPs. The particle size distribution histogram (Fig. 3F ) revealed that the produced EuM-NPs were within the nanoscale range, with particle diameters varying from approximately 1.23 to 26 nm. Analysis of 116 individual particles revealed a mean diameter of 15.64 ± 4.576 nm. The majority of nanoparticles were concentrated within the range of 12–20 nm, with the highest frequency observed around 15–16 nm, suggesting efficient control of particle growth during the synthesis process. The obtained particle size is considerably smaller than that reported for natural Sepia eumelanin, which typically consists of spherical granules with diameters of approximately 150 nm^50,51^, and is also below the general size range of melanin particles (60–80 nm) reported by Pralea et al.^52^. Likewise, Mbonyiryivuze et al.^53^ observed aggregated Sepia melanin particles composed of spherical granules ranging from 100 to 200 nm. In contrast, the average size of the present EuM-NPs is comparable to or smaller than several previously reported synthetic and biologically derived melanin nanoparticles. Liopo et al.^54^ reported melanin-like nanoparticles with an average size of 48 ± 12 nm. While Lemaster et al.^55^ synthesized ultrasmall eumelanin nanoparticles ranging from 9.4 to 31.4 nm under UV irradiation in weakly acidic and neutral conditions.

Similarly, Wang et al.^56^ described produced black particles of around 20 nm generated by *Vibrio natriegens*. Therefore, the EuM-NPs synthesized in the present study fall within the lower end of the reported size range for eumelanin-based nanoparticles. Such ultrasmall dimensions are particularly beneficial because they provide a high surface-area-to-volume ratio, which can enhance their physicochemical properties, thereby expanding the potential applicability of EuM-NPs in biomedical, pharmaceutical, catalytic, and nanotechnological fields.

Energy dispersive X-ray spectroscopy (EDX) analysis was carried out to reveal the elemental composition of the EuM-NPs. The EDX spectrum (Supplementary Fig. 2A) shows that EuM-NPs possesses carbon, oxygen, and nitrogen. TEM elemental mapping analysis results illustrate the whole distribution of the EuM-NPs and its components (C, N, O) (Supplementary Fig. 2B–E).

#### ζ-potential analysis

**Fig. 4:**
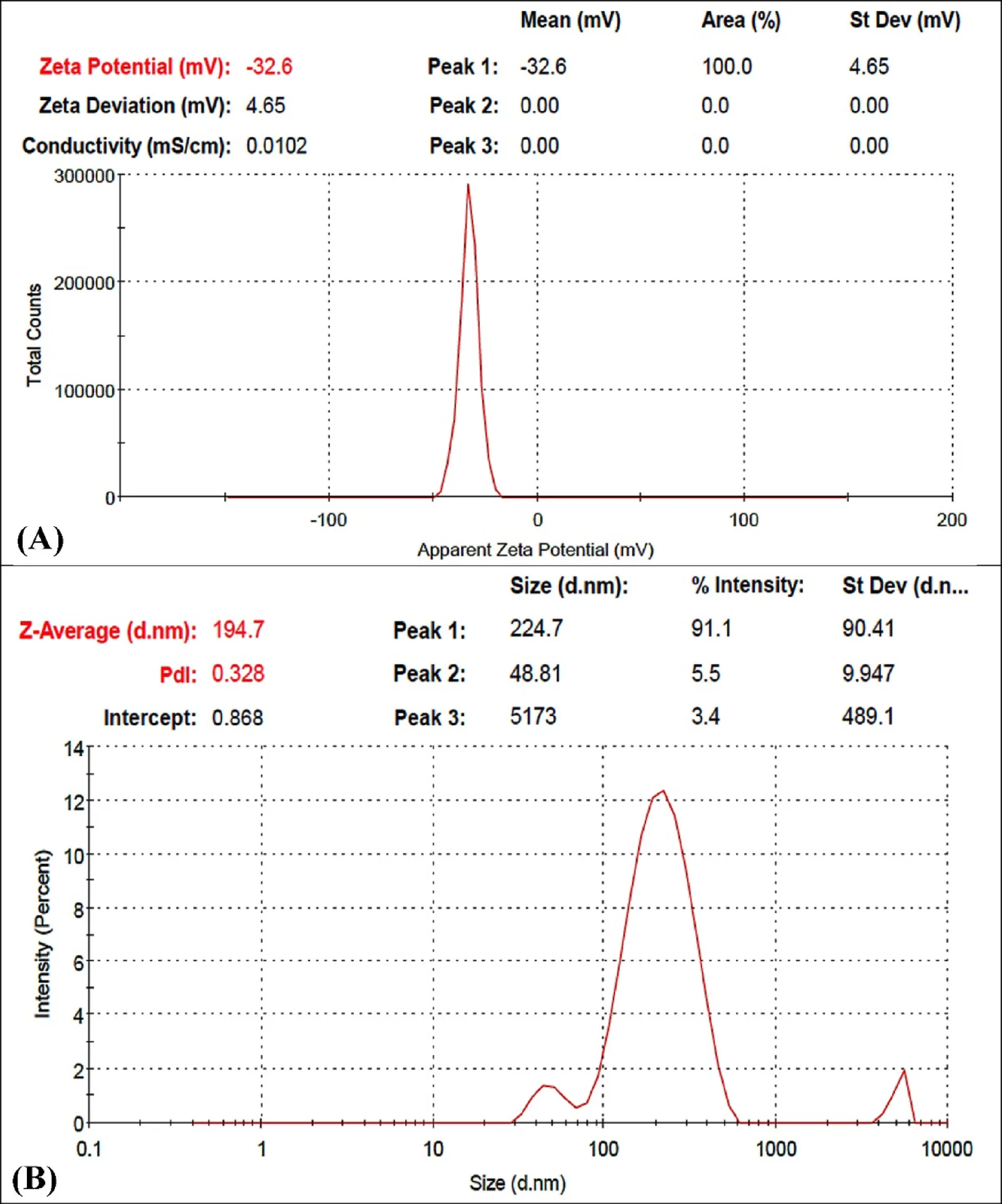
Characterization of EuM-NPs produced by *Streptomyces glaucescens* NEAE-H. (**A**) Zeta potential distribution showing zeta potential of − 32.6 mV. (**B**) Particle size distribution determined by dynamic light scattering showing a dominant peak at 224.7 d.nm (Z-average = 194.7 d.nm) with a polydispersity index of 0.328.

The ζ-potential of the synthesized EuM-NPs produced by *Streptomyces glaucescens* NEAE-H was determined to be − 32.6 ± 4.65 mV (Fig. 4A). The negative zeta potential of − 32.6 mV indicates that the EuM-NPs produced by *Streptomyces glaucescens* NEAE-H possess a negatively charged surface. A zeta potential magnitude greater than ± 30 mV is generally considered indicative of good electrostatic stabilization, which prevents particle aggregation and promotes colloidal stability^57^. The measured zeta potential of − 32.6 mV indicating that the EuM-NPs are adequately stabilized in aqueous suspension and are expected to resist aggregation due to electrostatic repulsion between particles. The electrophoretic analysis revealed a single, well-defined peak with 100% of particles contributing to Peak 1, indicating a homogeneous population. The zeta deviation of 4.65 mV reflects good reproducibility and stability of the measurement. The stability parameters determined indicate that the EuM-NPs are suitable for further applications in biomedical imaging, drug delivery systems, antioxidant therapeutics, and environmental remediation without requiring additional stabilizing agents or surface functionalization.

Recombinant melanin nanoparticles (RMNPs) prepared by the bottom-up nanocrystallization approach exhibited a particle size of 245.9 ± 31.5 nm and a zeta potential of − 20.2 ± 1.56 mV. In comparison, RMNPs produced using the double emulsion–solvent evaporation method showed a particle size of 253.1 ± 30.6 nm and a zeta potential of − 39.2 ± 0.56 mV, whereas those fabricated by high-pressure homogenization exhibited a larger particle size of 302.2 ± 69.9 nm and a zeta potential of − 38.6 ± 2.25 mV^58^. The nanoparticles exhibited a zeta potential of − 41.63 mV. This value indicates a highly negatively charged surface and suggests good colloidal stability^59^.

#### Particle size analysis

The particle size distribution analysis revealed a Z-average hydrodynamic diameter of 194.7 d.nm (Fig. 4B). The Z-average hydrodynamic diameter of 194.7 d.nm represents the EuM-NPs dimensions as determined by dynamic light scattering, which measures the overall hydrodynamic volume including any adsorbed biomolecules or solvation layer surrounding the EuM-NPs. The intensity-weighted size distribution exhibited a dominant peak at 224.7 d.nm (91.1% intensity), which represents the primary population of nanoparticles. Two minor peaks were also detected at 48.81 d.nm (5.5% intensity) and 5173 d.nm (3.4% intensity), though these represent a negligible fraction of the sample.

The polydispersity index (PDI) was 0.328, indicating a relatively narrow size distribution characteristic of a moderately monodisperse sample. Emulsions were classified as stable when the PDI was below 0.5, reflecting an acceptable degree of monodispersity, and the absolute ζ-potential exceeded 30 mV, indicating adequate electrostatic repulsion to minimize flocculation under the investigated ionic strength conditions^57^. Dynamic light scattering (DLS) analysis of sepia eumelanin particles revealed a hydrodynamic diameter distribution ranging from 180 to 260 nm^60^.

The particle size obtained by dynamic light scattering (DLS) was larger than that measured by scanning electron microscopy (SEM) or transmission electron microscopy (TEM), as DLS determines the hydrodynamic diameter, which includes the solvation or hydration shell surrounding the particles as well as any associated other molecules. In contrast, SEM and TEM provide the dimensions of the dehydrated particle core from dried samples. Accordingly, DLS reflects the apparent size of particles in their native aqueous environment as solvated, dynamically dispersed entities, whereas electron microscopy techniques represent their solid-state morphology^60^. Consequently, hydrodynamic diameters obtained by DLS are typically larger than those derived from SEM or TEM analysis.

#### TGA of EuM-NPs

**Fig. 5:**
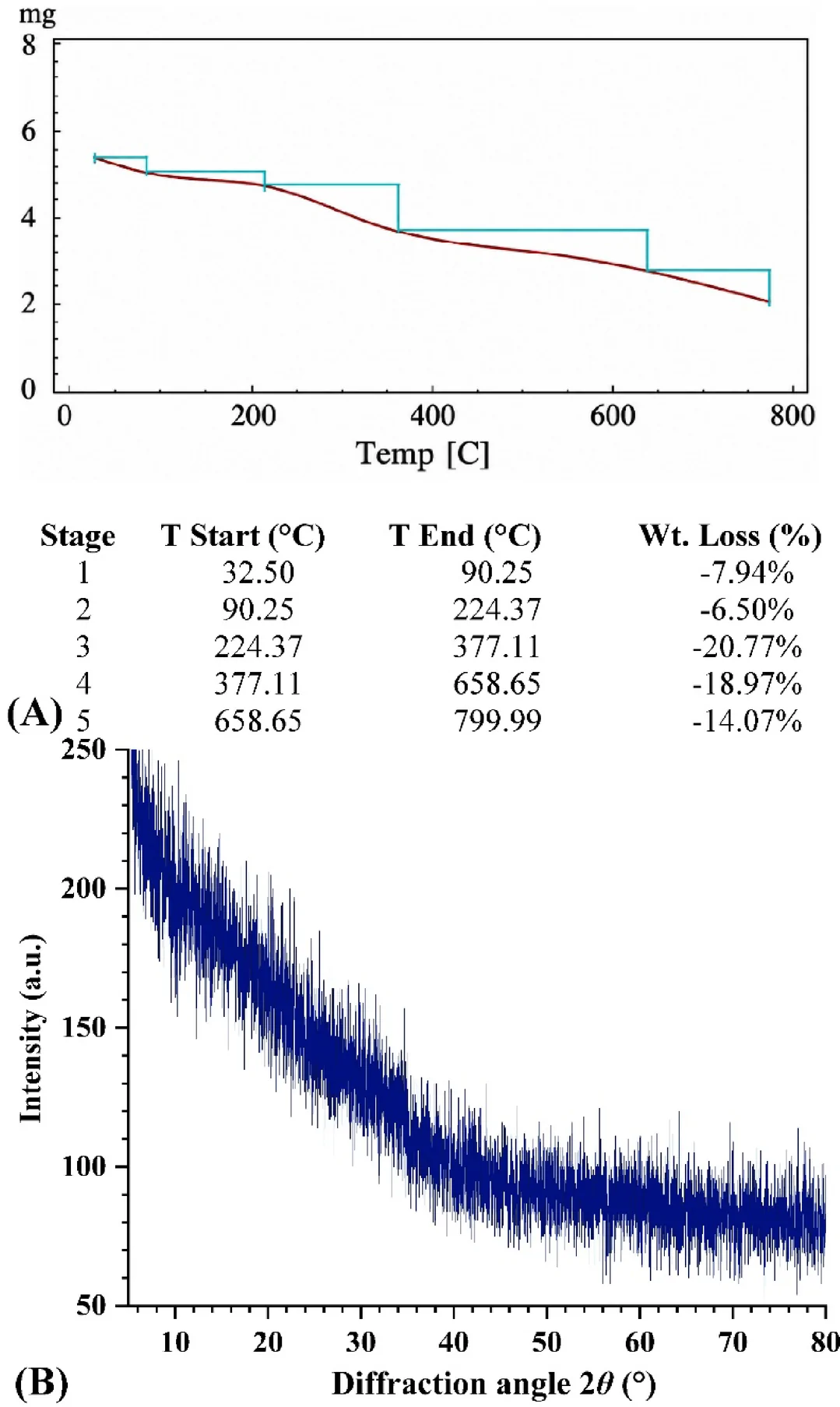
(**A**) Thermogravimetric analysis of EuM-NPs, illustrating the successive thermal decomposition stages and weight-loss profile under increasing temperature. (**B**) X-ray diffraction pattern of the synthesized EuM-NPs.

The thermal stability of EuM-NPs was investigated using TGA over the temperature range of room temperature to 800 °C (Fig. 5A). The TGA curve of EuM-NPs exhibited five distinct decomposition stages distributed across the temperature range of 32.5 to 800 °C. The initial sample mass of 5.403 mg progressively decreased through these stages.

The first weight-loss stage occurred between 32.50 and 90.25 °C, resulting in a mass loss of 7.94% (0.429 mg). This initial reduction is attributed to the evaporation of physically adsorbed water and loosely bound moisture trapped within EuM-NPs structure. The hygroscopic nature of eumelanin facilitates water retention through hydrogen bonding interactions, leading to the observed mass loss at relatively low temperatures. The initial endothermic peak was primarily attributed to the evaporation of weakly and/or strongly bound water molecules^52^. The initial mass-loss peak observed at 77.43 °C was primarily attributed to the evaporation of weakly bound water molecules^61^.

The second degradation stage, observed between 90.25 and 224.37 °C, accounted for an additional weight loss of 6.50% (0.351 mg). This stage represents the loss of organic compounds and weakly bound functional groups located on the EuM-NPs surface and any remaining organic residues from the synthesis process. The relatively limited mass reduction within this region indicates that the EuM-NPs structure remains largely intact. The second exothermic peak was primarily attributed to the loss of carbon dioxide^52^.

The most pronounced decomposition stage occurred between 224.37 and 377.11 °C, where a weight loss of 20.77% (1.122 mg) was recorded. This major degradation event is attributed to the breakdown of the eumelanin polymeric backbone, as well as decomposition of side-chain functionalities. The mass reduction is consistent with previous reports describing the thermal degradation behavior of microbial eumelanin. Melanin exhibited a gradual reduction in mass as the temperature increased from room temperature to 1000 °C. Approximately 25% of its initial weight was lost at 285.4 °C^62^. The thermal decomposition stage, occurring at 319.72 °C, corresponded predominantly to carbon dioxide evolution and yielded a residual mass of 72.86%^61^.

The fourth decomposition stage extended from 377.11 to 658.65 °C, resulting in a weight loss of 18.97% (1.025 mg). This prolonged decomposition reflects secondary thermal decomposition of aromatic components and carbonaceous intermediate compounds formed during the earlier stages of thermal decomposition processes. The fifth and final decomposition stage occurred between 658.65 and 799.99 °C, accompanied by a weight loss of 14.07% (0.760 mg). This stage was primarily attributed to decarboxylation reactions, which are recognized as the major thermal degradation processes of melanin at elevated temperatures^52^.

Similar findings have been reported for eumelanin, where a 50% weight loss was observed at 692.5 °C, and only 15% of the original mass remained at 1000 °C, demonstrating substantial thermal degradation while confirming the high thermal stability of melanin over a broad temperature range^62^. Likewise, a major thermal degradation stage at 754.73 °C was attributed to decarboxylation processes, leaving a residual mass of 43.36% at 850 °C. Previous studies have shown that the aliphatic components of melanin decompose at temperatures below 400 °C, while the more thermally stable aromatic components undergo decomposition at temperatures exceeding 400 °C^61^.

The cumulative weight loss of EuM-NPs reached approximately 68.24%, leaving a residual mass of 31.76% at 800 °C. These TGA results demonstrate the excellent thermal stability of EuM-NPs at elevated temperatures, highlighting their multistage decomposition with 31.76% residual mass. The observed thermal behavior supports the suitability of EuM-NPs for applications requiring high thermal stability, including biomedical, pharmaceutical, antioxidant, coating, and nanotechnological applications.

#### XRD analysis of EuM-NPs

The X-ray diffraction pattern of the produced EuM-NPs is presented in Fig. 5B. The diffractogram exhibited a broad diffuse halo over the investigated 2*θ* range of 5–80°, without the appearance of sharp and well-defined diffraction peaks. The diffraction intensity gradually decreased with increasing diffraction angle, and no distinct diffraction peaks corresponding to crystalline phases were detected. These findings indicate that the produced EuM-NPs possess a predominantly amorphous structure rather than a highly crystalline arrangement. The observed amorphous nature of the EuM-NPs is consistent with the intrinsic structural characteristics of eumelanin. Eumelanin consists of heterogeneous oligomeric assemblies derived from indole-based monomeric units, primarily 5,6-dihydroxyindole (DHI) and 5,6-dihydroxyindole-2-carboxylic acid (DHICA). The irregular stacking of these oligomers, together with extensive intermolecular interactions mediated by hydrogen bonding and π–π stacking among the aromatic moieties, prevents the development of long-range crystallographic order. Therefore, the absence of sharp diffraction peaks in the present study confirms that the produced EuM-NPs retained the characteristic amorphous architecture of eumelanin.

### LC–MS/MS analysis

**Fig. 6:**
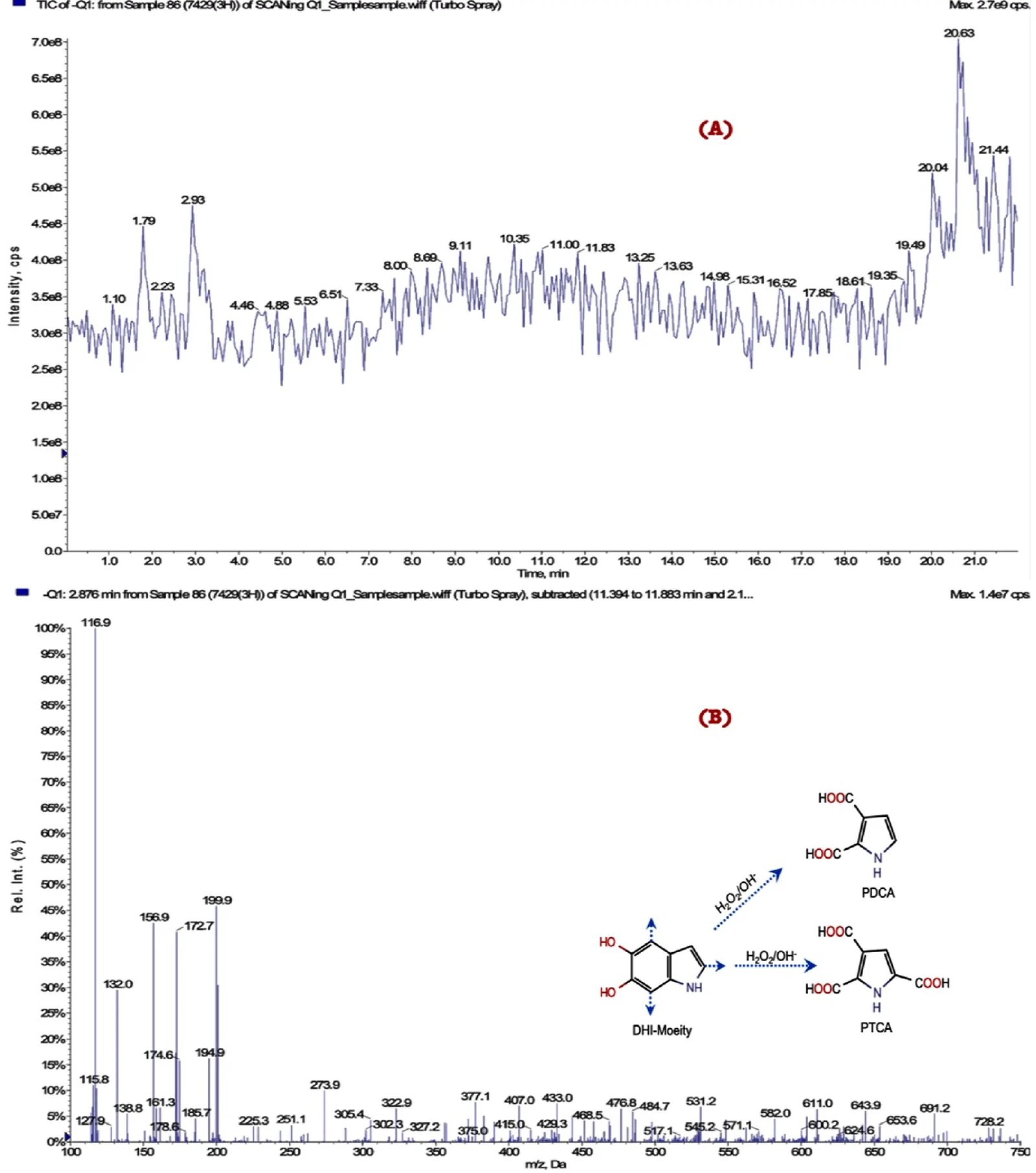
LC–MS/MS analysis (**A**) Total Ion Current (TIC) Chromatogram. (**B**) Ion product spectra for the [M + H] + molecular ions of EuM-NPs oxidative fragments PDCA [*m/z* 156.9] and PTCA [*m/z *199.9].

Structural elucidation of EuM-NPs produced by *Streptomyces glaucescens* strain NEAE-H was further characterized by LC–MS/MS analysis. The total ion chromatogram (TIC) displayed a complex profile with multiple peaks distributed across the entire elution window (1–21 min), with the most prominent signals appearing at retention times of 1.79, 2.93, 20.04, 20.63, and 21.44 min, reaching a maximum intensity of 2.7 × 10⁹ cps (Fig. 6). The broad, heterogeneous chromatographic profile is characteristic of eumelanin, which is not a discrete molecule but a complex heteropolymer composed of oligomeric and polymeric indole units of varying chain lengths and oxidation states. The mass spectrum acquired at 2.876 min (Fig. 6) revealed a series of fragment ions consistent with the core structural units of eumelanin. The base peak at *m/z* 116.9, along with prominent ions at *m/z* 132.0, 156.9, 172.7, 174.6, 194.9, and 199.9, corresponds to low-molecular-weight indole-derived monomeric and dimeric fragments generated upon ionization of the eumelanin polymer. Higher-mass ions detected at *m/z* 273.9, 322.9, 377.1, 433.0, 484.7, 531.2, 582.0, 643.9, and 691.2 are consistent with oligomeric eumelanin units of increasing degree of polymerization.

Eumelanin and pheomelanin degradable byproducts can be analytically quantified using liquid chromatography^52,63,64^. LC–MS analysis performed in positive-ion mode revealed a prominent ion at *m/z* 433. However, because eumelanin is a structurally heterogeneous polymer, this ion should not be interpreted as representing a definitive molecular weight for the entire eumelanin polymer. Assuming that the detected ion corresponds to [M+H]⁺, its tentative neutral molecular mass was estimated to be approximately 432 Da.

Considering the melanin polymeric nature, subtypes, harsh treatments, preparation method, and analysis conditions, the reported data would rather be heterogenous, and no exact *m/z* distribution can be assigned. Alkaline H_2_O_2_ oxidation is a more efficient and less labor-intensive oxidative procedure applicable to melanin-containing samples^52,65–67^. In the current LC–MS/MS estimation, ammonium hydroxide was used for EuM-NPs alkalization. LC–MS/MS positive ion mode [M^+^H]^+^ transition patterns (Fig. 6B) represent the product ion spectra for the oxidative degradable markers of EuM-NPs hydrolysis, pyrrole‐2,3‐dicarboxylic acid (PDCA) [*m/z* 156.9], and pyrrole‐2,3,5‐tricarboxylic acid (PTCA) [*m/z *199.9]. PDCA fragment has been previously monitored at negative ion mode transition [M-H]- of *m/z* 154.13 -> 110.13 and *m/z* 154.13 -> 66.15 in human skin biopsies^68^. While the negative ion mode transition [M-H]- of the PTCA fragment was also been monitored at *m/z* 198 -> 154 and *m/z* 198 -> 110 in both human skin biopsies^67,68^ and hair samples^69,70^.

On the other hand, non-alkaline oxidative sample LC–MS/MS preparation methods were unable to clearly differentiate the possible monomers, however, eumelanin dissolved in DMSO showed peak patterns of two forms (*m/z* 475.5 and *m/z* 701.5), with the second peak being considered a dimeric form^52^. Thus, PDCA and PTCA fragments are considered quantifiable oxidative markers of EuM-NPs in oxidized samples. Therefore, the detection of these compounds by LC–MS/MS supports the eumelanin-like chemical composition of EuM-NPs.

### In vivo antitumor Ehrlich solid tumor model

#### Mean survival time (MST) and percentage increase in life span (% ILS)

The tumor-bearing mice injected with Dox or EuM-NPs were monitored, and the days of survival were documented. In comparison with EAC control mice, Dox treatment significantly raised the survival time from 17.2 ± 2.4 to 49.7 ± 4.1 days. Also, the survival time for EuM-NPs-treated mice has significantly increased in a dose-dependent manner from 17.2 ± 2.4 to 38.8 ± 3 days, from 17.2 ± 2.4 to 45.1 ± 1.3 days, and from 17.2 ± 2.4 to 56.3 ± 2.8 days in EAC/ EuM-NPs 5, EAC/ EuM-NPs 10, and EAC/ EuM-NPs 20 groups; respectively; compared to EAC control group (Table 2).

**Table 2:** Effect of EuM-NPs treatment on survival of mice bearing solid tumor.

Groups	MST (day)	ILS (%)
EAC	17.2 ± 2.4	–
DOX	49.7 ± 4.1*	288.95
EAC/ EuM-NPs 5	38.8 ± 3.0*	225.58
EAC/ EuM-NPs 10	45.1 ± 1.3*	262.20
EAC/ EuM-NPs 20	56.3 ± 2.8*^$^	327.32

Data are expressed as means ± SEM (n = 3). Statistical comparisons were made using one way of analysis of variance (ANOVA) followed by Tukey–Kramer post hoc test for multiple comparisons. *Represents significant difference vs. EAC at *P*< 0.05; $ represents significant difference vs. EAC/EuM-NPs 5 at *P*< 0.05

#### Tumor’s volume and weight determination

**Fig. 7:**
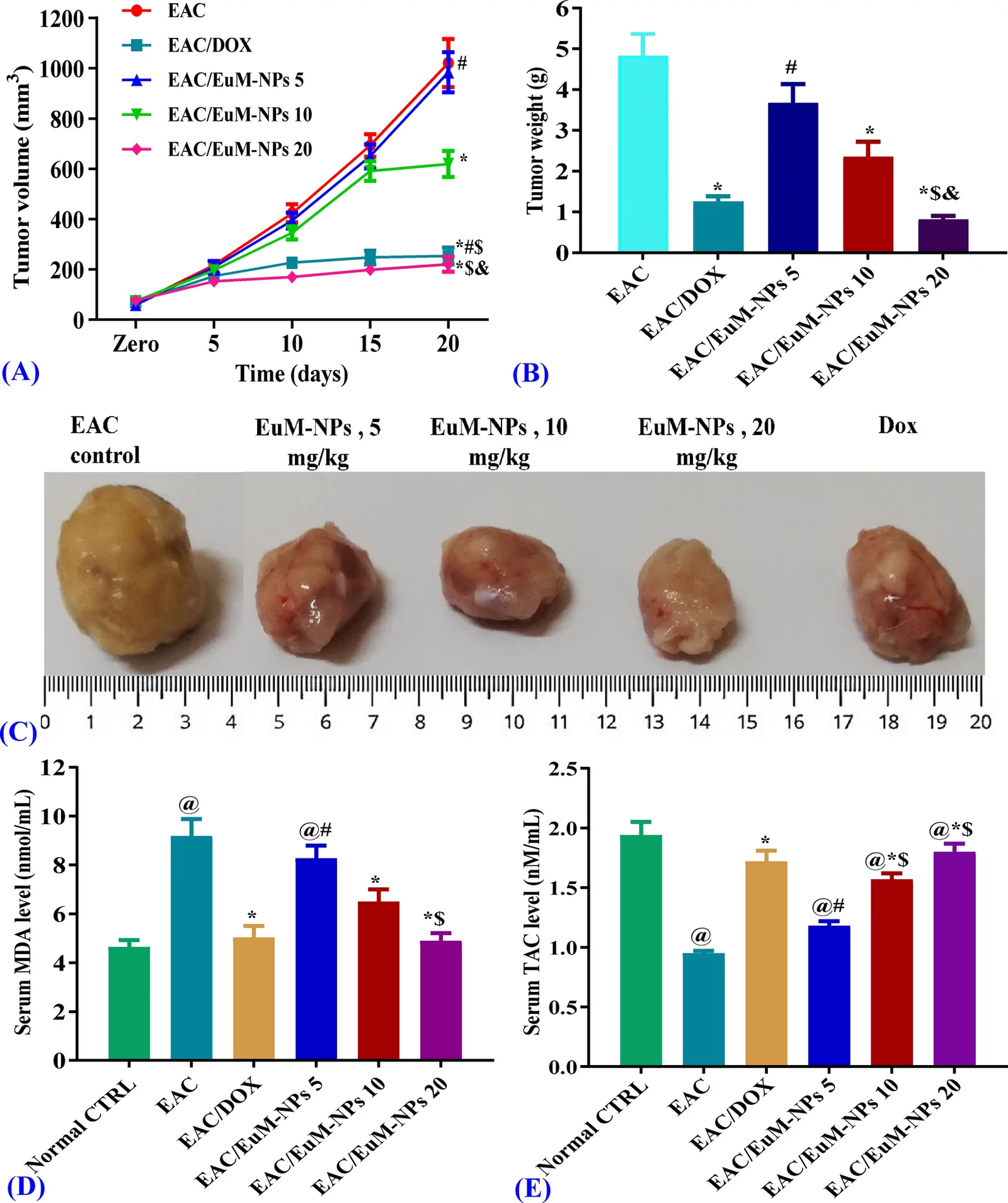
Effect of EuM-NPs on tumor volume (**A**) and tumor weight (**B**), and representative photographs of excised tumors (**C**) of EAC bearing mice. Effects of EuM-NPs on serum MDA (**D**) and TAC (**E**) levels of EAC bearing mice. Data are expressed as means ± SEM (n = 5). Statistical comparisons were made using one way of analysis of variance (ANOVA) followed by Tukey–Kramer post hoc test for multiple comparisons. * Significant difference vs. EAC CTRL at *P* < 0.05; # Significant difference vs. EAC/DOX at *P* < 0.05; $ significant difference vs. EAC/ EuM-NPs 5 at *P* < 0.05, & Significant difference vs. EAC/ EuM-NPs 10 at *P* < 0.05; @ represents significant difference vs. normal CTRL at *P * < 0.05 . All tumor images were taken at the same magnification power, zooming, and distance from the camera.

Tumor volume assessment every 5 days throughout the experiment revealed a significant decrease in tumor growth, where the tumor volume in the EAC group raised from 63.4 ± 10.4 to 1021.9 ± 96.3 mm^3^ on the 21^st^ day. Compared with EAC mice on the 21st day, DOXO treatment inhibited the tumor growth by 75%. Moreover, mice in the EAC/ EuM-NPs 5, EAC/ EuM-NPs 10, and EAC/ EuM-NPs 20 groups showed a significant decrease in tumor weight by 9.6%, 40%, and 78%, respectively, compared to the EAC group demonstrating a significant antitumor action of EuM-NPs in mice bearing Ehrlich solid tumor (Fig. 7).

Estimation of Ehrlich solid tumor weight on the 21st day demonstrated that DOX decreased the tumor weight by 73.2%, while EuM-NPs (5 mg/kg) decreased tumor weight by 24% in comparison with the EAC group. In addition, mice in the EAC/ EuM-NPs 10 and EAC/ EuM-NPs 20 groups showed a decrease in tumor weight by 51% and 83%, respectively, compared to the EAC group (Fig. 7). The significant tumor reduction in the EuM-NPs -injected group highlights the significant dose-dependent antitumor efficacy of EuM-NPs.

#### Lipid peroxide (LPO) and total antioxidant capacity (TAC) evaluation

The untreated EAC group displayed a significant elevation in serum MDA concentration in comparison with the normal control group. However, serum MDA concentration was significantly reduced in EAC/DOX, EAC/ EuM-NPs 10, and EAC/ EuM-NPs 20 groups compared to EAC mice. Moreover, there was a more significant decline in serum MDA concentration in EAC/ EuM-NPs 10 and EAC/ EuM-NPs 20 groups compared to EAC/ EuM-NPs 5 (Fig. 7). In parallel, serum TAC level was significantly diminished in EAC mice in comparison with the normal control group. The EAC/DOX group displayed a significant increase in serum TAC level when compared to EAC-bearing mice. Also, EuM-NPs (10 and 20 mg/kg) treatment significantly ameliorated the EAC-induced decrease in serum TAC (Fig. 7).

Carcinogenesis is a multistep process including mutation and expanded cell proliferation. Overproduction of reactive oxygen species (ROS) and defective antioxidant and/or DNA repair mechanisms can cause oxidative stress that destroys cellular macromolecules. The released ROS can oxidize cellular fatty acids producing lipid peroxyl radicals and lipid hydroperoxides, which generate MDA^71^. The strong positive association between tumor size and circulating MDA and the negative one with TAC implied that the antioxidant activity of EuM-NPs may be one of the possible mechanisms of its anti-tumor impact. During tumor growth, cells that are continuously subjected to oxidative stress become very resistant and elaborate strong antioxidative protection behavior^72^. This antioxidant potential may be one of the explanations for EuM-NPs cytotoxicity against tumor cells observed in vivo.

#### Histopathological examination

**Fig. 8:**
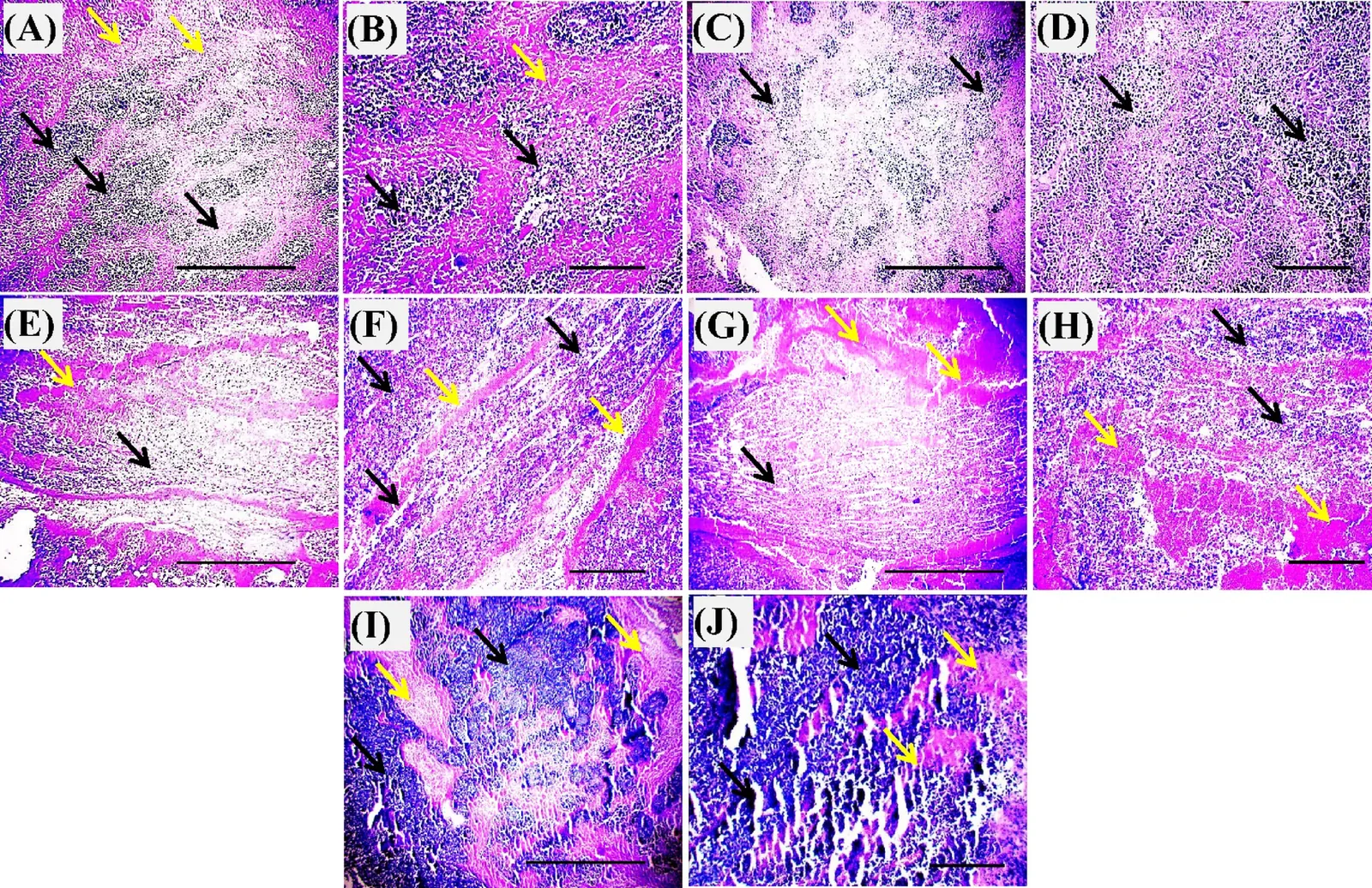
Histopathological analysis of mice bearing Ehrlich solid tumor. Microphotograph through growing tumor stained with H & E showing living tumor foci (black arrows) and areas of necrosis (yellow arrows). Areas of necrosis are wider in the EAC group (**A**, **B**) and EAC/EuM-NPs 5 group (**C**, **D**), decreasing gradually in the EAC/EuM-NPs 10 group (**E, F**) and then in the EAC/DOX group (**G**, **H**) followed by EAC/EuM-NPs 20 group (**I**, **J**). (**H&E**), X40 magnification, scale bar = 200 µm (**A**, **C**, **E**, **G**, **I**); X100 magnification, scale bar = 100 µm (**B**, **D**, **F**, **H**, **J**).

H&E-stained microscopic pictures of EAC mice revealing living tumor foci and areas of necrosis. Areas of necrosis are wider in the EAC group (Fig. 8A, B) and EAC/ EuM-NPs 5 group (Fig. 8C, D), decreasing gradually in the EAC/ EuM-NPs 10 group (Fig. 8E, F) then in the EAC/DOX group (Fig. 8G, H) followed by EAC/ EuM-NPs 20 group (Figs. 8I, J). The decreased necrosis observed in treated tumors should be considered a histopathological finding that may indicate altered tumor architecture and tissue preservation.

#### Immunohistochemistry analysis

**Fig. 9:**
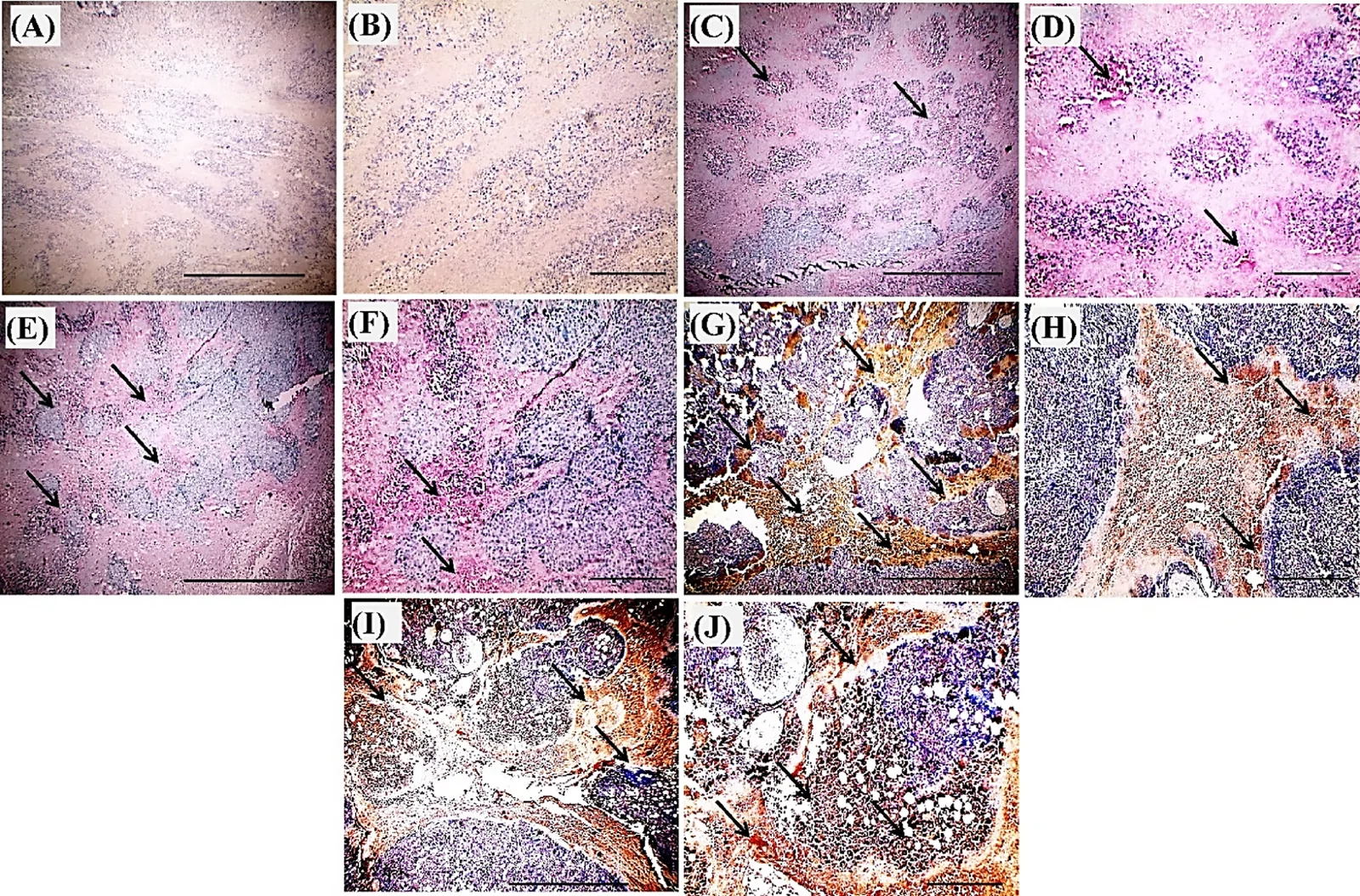
Immunohistochemistry of cleaved (active) caspase-3 in tumor sections. EAC mass immunostained against caspase-3 showing negative staining in the EAC group (**A**, **B**). The positive immunolabelling against caspase-3 (black arrows) appears very weak in the EAC/ EuM-NPs 5 group (**C**, **D**), weak in the EAC/ EuM-NPs 10 group (**E**, **F**), moderate in the EAC/DOX group (**G**, **H**), and strong in EAC/ EuM-NPs 20 group (**I**, **J**). IHC counterstained with Mayer’s hematoxylin, X40 mangnification, scale bar = 200 µm (**A**, **C**, **E**, **G**, **I**); X100 magnification, scale bar = 100 µm (**B**, **D**, **F**, **H**, **J**).

To verify whether the deterioration of tumor growth by EuM-NPs treatment was via apoptosis induction, we assessed the expression of active caspase-3 in tumor samples and observed that the EAC/ EuM-NPs 5 group showed very weak caspase-3 expression (Fig. 9C, D). While the positive immunolabelling against caspase-3 expression was mild in the EAC/ EuM-NPs 10 group (Fig. 9E, F). In the EAC/DOX group, the caspase-3 expression was moderate (Fig. 9G, H). Moreover, the caspase-3 expression was strong in the EAC/ EuM-NPs 20 group (Fig. 9I, J). Another possible mechanism explaining the melanin anti-tumor activity is its capacity to induce apoptosis in malignant cells. During apoptosis, cells are submitted to regulated cell death. Apoptosis resistance is a characteristic of cancer^73^. The two prominent extrinsic and intrinsic apoptotic pathways can activate caspase-3^74^, that supposed to be the effector-caspase in the accomplishment of apoptosis^75^. Consequently, we investigated the immunostaining of caspase-3 in tumor tissue and revealed that melanin significantly induced active caspase-3 immunostaining in a dose-dependent fashion. Although the observed modulation of oxidative stress markers and activation of caspase-3 suggest apoptosis-mediated cytotoxicity, further investigations involving upstream and downstream apoptotic biomarkers (e.g., Bax/Bcl-2 ratio, caspase-9 activation, PARP cleavage, and Annexin V/PI flow cytometry) are warranted to fully elucidate the molecular mechanisms underlying the anticancer activity of the produced EuM-NPs.

### In vivo antitumor Ehrlich ascites carcinoma model

#### Effect of EuM-NPs on hematological parameters

**Fig. 10:**
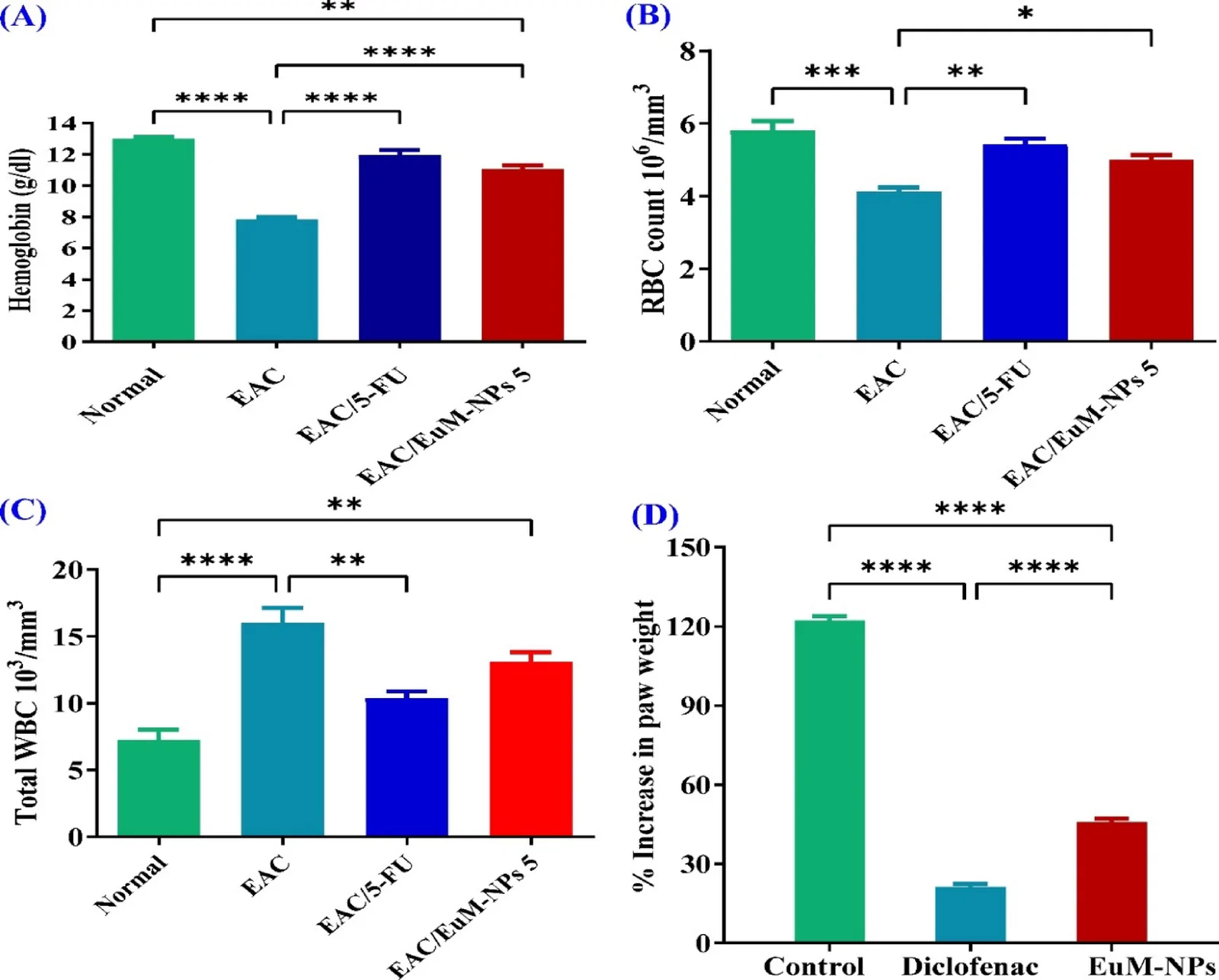
Effect of EuM-NPs on the hematological changes: (**A**) Hb content, (**B**) RBCs count, (**C**) WBCs count in Ehrlich ascites carcinoma model, and (**D**) effect of EuM-NPs on carrageenan-induced hind paw edema. Diclofenac (10 mg/kg) and EuM-NPs (10 mg/kg) were given 1 h before carrageenan injection. Measurement of paw weight was done 4 h after carrageenan injection. Data are expressed as means ± SEM. Statistical comparisons were made using one way of analysis of variance (ANOVA) followed by Tukey–Kramer post hoc test for multiple comparisons. *Significant difference at p < 0.05; **Significant difference at *P* < 0.01; ***Significant difference at *P* < 0.001; ****Significant difference at *P* < 0.0001.

The results showed that the EAC-bearing mice exhibit significant reduction in Hb content and RBCs count, together with a significant elevation in WBCs count, compared with the normal healthy control. Treatment with 5-FU or EuM-NPs (in EAC/5FU group and EAC/EuMNPs group, respectively) partially reversed these hematological alterations by increasing by increasing both Hb concentration and RBCs count as well as decreasing WBCs count relative to the untreated EAC group. (Fig. 10).

#### Effect of EuM-NPs on tumor burden and survival

The results revealed that treatment with 5-FU and EuM-NPs led to a significant reduction by 85% and 75%; respectively; in peritoneal ascites volume compared to the EAC-bearing mice (Table 3). Moreover, the treatment with 5-FU and EuM-NPs induced about 79% and 57%; respectively; a decrease in viable tumor cell count in comparison with the EAC-bearing mice. In addition, the non-viable tumor cell count was boosted in the EAC/5-FU and EAC/ EuM-NPs 5 groups by 6.4 and 4.9-folds; respectively; in comparison with the EAC group. However, MST of the EAC/ 5FU EAC/ EuM-NPs 5 groups was 47.5 and 34 days; respectively, while the EAC group exhibited 16.5 ± 1.4 days. The elevation in MST was 2.9- and 2.1-fold in the EAC/5-FU and EAC/ EuM-NPs 5 groups compared to the EAC group (Table 3).

**Table 3:** Effect of EuM-NPs treatment on tumor burden and survival in Ehrlich ascites carcinoma model.

Group	Tumor volume (mL)	Viable tumor cells count (10^6^)	Non-viable tumor cell count (10^6^)	MST (days)	% ILS
EAC group	8.9 ± 0.68	87.25 ± 4.1	12.75 ± 2.4	16.5 ± 1.4	–
EAC/5-FU	1.3 ± 0.21^#^	18.16 ± 2.1^#^	81.84 ± 3.9^#^	47.5 ± 2.6^#^	187.9%
EAC/ EuM-NPs 5	2.17 ± 0.17^#^	37.49 ± 2.8^#$^	62.51 ± 3.2^#$^	34 ± 2.3^#$^	106.1%

Data are expressed as means ± SEM (n = 5 for tumor volume and cell counts, n = 3 for MST and %ILS). Statistical comparisons were made using one way of analysis of variance (ANOVA) followed by Tukey–Kramer post hoc test for multiple comparisons.

#significant difference vs. EAC at *P* < 0.05; $ represents significant difference vs. EAC/5-FU at *P* < 0.05.

#### In vivo anti-inflammatory carrageenan-induced rat hind paw edema model

Inflammation is a principal physiologic guard mechanism that promotes the body to guard itself against infection, burn, toxic chemicals, allergens, or other noxious stimuli. Presently used anti-inflammatory medications have some serious adverse events. Hence, there is a crucial demand for the evolution of efficient anti-inflammatory drugs from medicinal plants origin that have less adverse events. Edema means swelling arises when part of the body becomes swollen because of abnormally large fluid accumulation and retention in the body’s tissues within spaces between the cells (interstitial spaces), which can cause severe pain. Although edema can distress any part of your body, it’s most observed in the hands, arms, feet, ankles, and legs. Cyclooxygenase-2 (COX-2) has an important role in the inflammation pathway through stimulation of the prostaglandins (PGs) synthesis. Carrageenan-induced inflammation in the rat paw is a conventional model of edema and hyperalgesia, which has been broadly utilized in the assay of the nonsteroidal anti-inflammatory drugs and selective cyclooxygenase-2 (COX-2) inhibitors^76^.

Sub-plantar administration of carrageenan into the rats′ hind paw caused a marked increase in paw weight. After 4 h of carrageenan injection, the percentage increase in paw weight was 122.26% for the vehicle-treated animals. Treatment of animals with diclofenac or EuM-NPs significantly inhibited the carrageenan-induced hind paw edema by 82.54 and 62.62%, respectively, compared to the control group. However, there were significant differences in paw edema reduction by EuM-NPs and the reduction produced by diclofenac (Fig. 10).

#### In vitro cyclooxygenases’ (COX-1/2) kinetics

EuM-NPs were examined for inhibitory effect on COX-1 and COX-2 enzymes in comparison with celecoxib as a standard selective COX-2 inhibitor. As demonstrated in Table 4, celecoxib and EuM-NPs showed significant anti-inflammatory potential at COX-2 enzyme at the used concentrations. When the selectivity ratio is greater than 1.0, the NSAIDs are thought to be more potent COX-2 inhibitors^77^. From the IC_50_ values and selectivity ratios, we can realize that EuM-NPs have a higher selectivity for COX-2 over COX-1, with a selectivity ratio of > 8.91, indicating that EuM-NPs could act as a selective COX-2 inhibitor and inhibition of COX-2 by EuM-NPs may be a possible mechanism for its anti-inflammatory activity.

**Table 4:** The in vitro COX-1/COX-2 enzyme inhibition assay of the EuM-NPs.

Comp	IC_50_^a^		Selectivity index (SI)^C^
	COX-1	COX-2	
Celecoxib	> 50(µg/mL)^b^	0.34 (µg/mL)	> 147.05
EuM-NPs	> 50 µg/mL)^b^	5.61	> 8.91

^a^IC_50_ value is the compound concentration required to produce 50% inhibition of COX-1 or COX-2 for means of two determinations and deviation from the mean is < 10% of the mean value; ^b^No inhibition of COX-1 up to 50 µg/mL; ^c^Selectivity index (COX-1 IC_50_/COX-2 IC_50_).

The expression of COX-1 is constitutive in utmost tissues and is included in keeping normal physiological processes, such as platelet aggregation, gastric mucosal protection, and control of blood flow to the kidneys, while the expression of COX-2 is initiated due to proinflammatory stimulants and is mostly responsible for the creation of pain-producing prostaglandins^78^. Also, the COX-2 enzyme is universally overexpressed in a variety of human malignancies, and previous studies have repetitively confirmed that drugs that inhibit COX-2 can reduce tumor progression and metastasis in different cancer models^79^. In addition, some investigations have proposed that COX inhibitors, specifically COX-2 inhibitors, may have anticancer activities and might be applied as anticancer agents^80^.

### In silico computational analysis

#### Drug-likeness and pharmacokinetics (ADME/Tox) analysis

Despite the recent growing interest in eumelanin’s biological potential, until now there is no satisfactory understanding of its exact macromolecular structure, and the connectivity manner between its cross-linked building blocks is still controversial^81^. Thus, seven of the most probable precursors of 5,6-dihydroxyindole (DHI)-based mono/oligo/polymeric redox fragments (Supplementary Fig. 3) have been selected from literature^82–90^ for in silico virtual biological investigation.

**Fig. 11:**
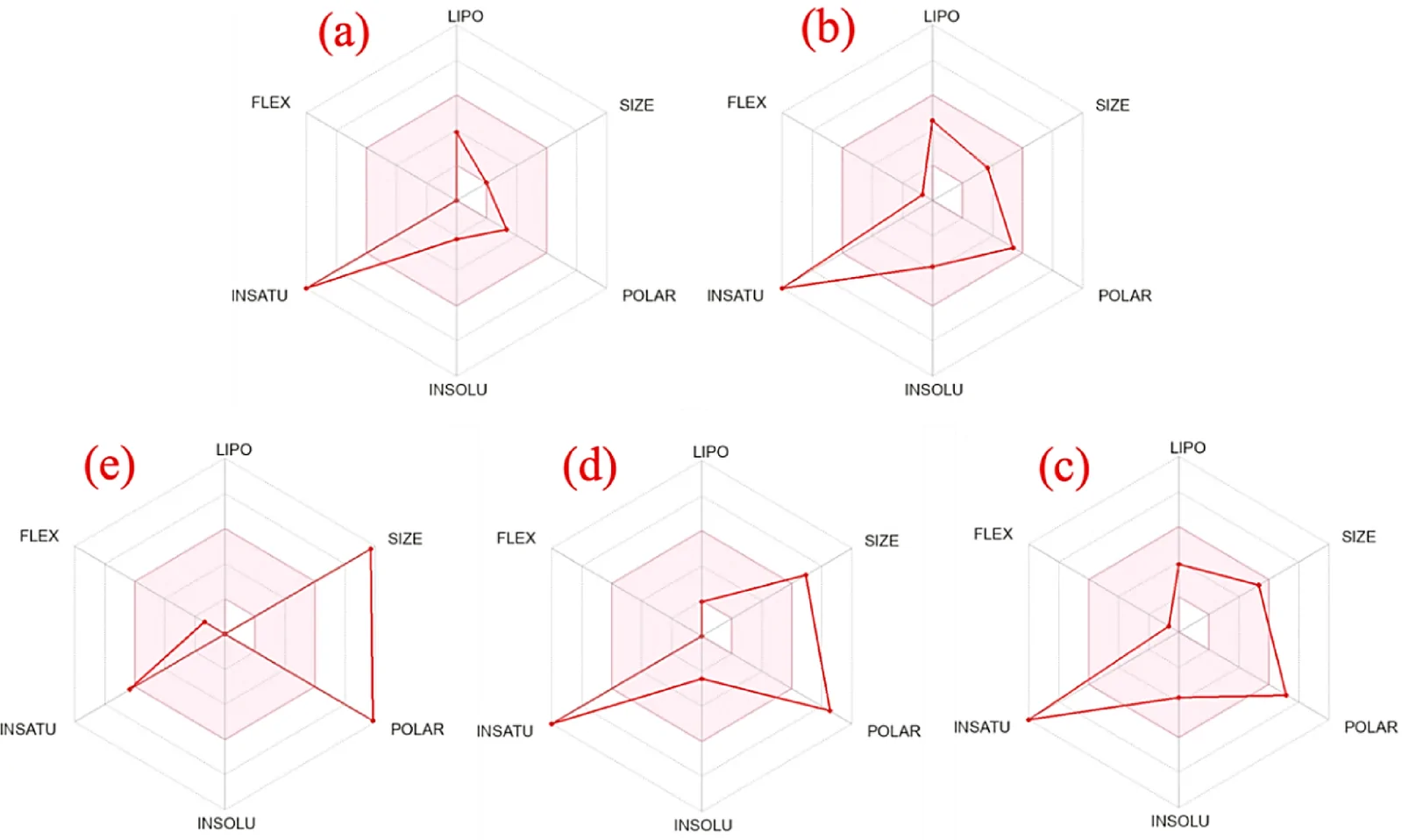
Bioavailability Radar for pharmacokinetics, drug-likeness, and oral bioavailability assessment of eumelanin and its mono/oligo/polymeric precursors. (**a**) DHI: 5,6-dihydroxy-indole; (**b**) DHI-dimers: 2,2′-biindolyl (2,2′ DHI-dimer), 2,4′-biindolyl (2,4′ DHI-dimer) and, 2,7′-biindolyl (2,7′ DHI-dimer) (**c**) Linear DHI-trimer; (**d**) macrocyclic DHI-tetramer; (**e**) Polycyclic DHI-pentamer eumelanin. LIPO: lipophilicity (XLOGP3); SIZE: molecular weight (MW); Polar: polarity (topological polar surface area ‘TPSA’); INSOLU: insolubility in water (logS); INSATU: insaturation of carbons in the sp3 hybridization; FLEX: flexibility as a number of rotatable bonds.

ADME/Tox prediction profile (Table 5) including molecular descriptors, and drug-likeness, pharmacokinetics of the most investigated 7-DHI-based fragments reflects their structural complexity that might be attributed to their protective shielding efficacy from the deleterious UV penetration through the skin by absorbing and/or partially scattering ultraviolet light^91^. This protective shielding barrier might be desirable for some industrial applications including cosmetic and skincare formulations. As shown in Table 5, almost all of the tested DHI-based oligo/polymeric fragments wouldn’t obey the Lipinski role of five^92^, because of their structural complexity. Among them, the monomeric DHI precursor would be predicted as the only appropriate drug-like candidate without violating the Lipinski role of five, reflecting a good oral bioavailability (Fig. 11a) with a bioavailability score of 0.55, higher percentage absorption (ABS%) of 89.5938%, and higher topological polar surface Area (TPSA) of 56.25 Å^2^.

**Table 5:** Comparative drug-likeness and pharmacokinetics (ADME/Tox) parameters and molecular descriptors of (DHI)-based eumelanin mono/oligo/polymeric precursors:

	DHI	2,2′ DHI dimer	2,4′ DHI dimer	2,7′ DHI dimer	DHI trimer	DHI tetramer	Polycyclic Eumelanin
*Drug-likeness parameter*							
Mwt. (g/mol)	149.15	296.28	296.28	296.28	441.39	580.46	823.76
HBDs	3	6	6	6	7	4	9
HBAs	2	4	4	4	6	8	14
RotBs	0	1	1	1	1	0	2
TPSA (Å2)	56.25	112.5	112.5	112.5	162.43	199.72	235.72
log S (ESOL)	− 2.21	− 3.76	− 3.74	− 3.74	− 3.72	− 2.45	0.98
Class	Soluble	Soluble	Soluble	Soluble	Soluble	Soluble	Highly soluble
Log Po/w (MLOGP)	0.3	0.45	0.45	0.45	− 1.39	− 3.47	− 1.12
nV	0	1	1	1	1	2	3
%ABS (%)	89.5938	70.1875	70.1875	70.1875	52.9617	40.0966	27.6766
Bio-availability score	0.55	0.55	0.55	0.55	0.55	0.17	0.17
*Pharmacokinetics*							
GI-Absorption	High	High	High	High	Low	Low	Low
BBB permeant	Yes	No	No	No	No	No	No
Log Kp (cm/s) (skin permeation)	− 6.26	− 6.37	− 6.39	− 6.39	− 8.11	− 11.34	− 17.86

Physicochemical properties calculated on SwissADME: MW: molecular weight; RotBs: rotatable bonds; HBDs: hydrogen bonding donor; HBAs: hydrogen bonding acceptor; MlogP (Moriguchi Log P): octanol/water partition coefficient (Log Po/w); nV: number of Lipinski violations; TPSA: total polar surface area; LogS: coefficient of solubility determined by the ESOL method; Class: insoluble < − 10 < poor < − 6 < moderately < − 4 < soluble < − 2 < very < 0 < highly; %ABS was expressed by the equation %ABS = 109 − (0.345 × TPSA).

The score of all compounds concerning the drug score was determined by combining records of similarity with already approved drugs, such as lipophilicity, solubility, molecular mass, and toxicity risks, and a single numeric value was given, which ranges from 0.0 to 1.0 and can be used to predict the global potential of a compound as a new drug candidate.

It also demonstrated enough lipophilicity to enhance its gastrointestinal absorption (HIA) and blood–brain barrier (BBR) permeability (Supplementary Fig. 4). DHI-dimers (2,2′ DHI dimer, 2,4′ DHI dimer, and 2,7′ DHI dimer) showed good gastrointestinal absorption and poor BBR permeability. While the macro-fragments including DHI-based trimer, tetramer, and polycyclic pentamer showed poor efficacy for both gastrointestinal absorption and BBR permeability (Supplementary Fig. 4). A moderate oral bioavailability (Fig. 11b) was shown for the three DHI-dimers with ABS% of 70.1875%, and TPSA of 112.5Å^2^. While the macro-fragments showed a relatively poor bioavailability (Fig. 11c, d, e).

Both DHI and DHI-dimers showed a relatively moderate potential for skin barrier permeability with a skin permeation coefficient (Log K*p*) ranging from − 6.26 to − 6.39 cm/s. While DHI-trimer, tetramer, and polycyclic pentamer showed a significant to extreme lower skin permeability with Log K*p* of − 8.11, − 11.34, and − 17.86 cm/s, respectively.

#### Network pharmacology and target prediction

**Fig. 12:**
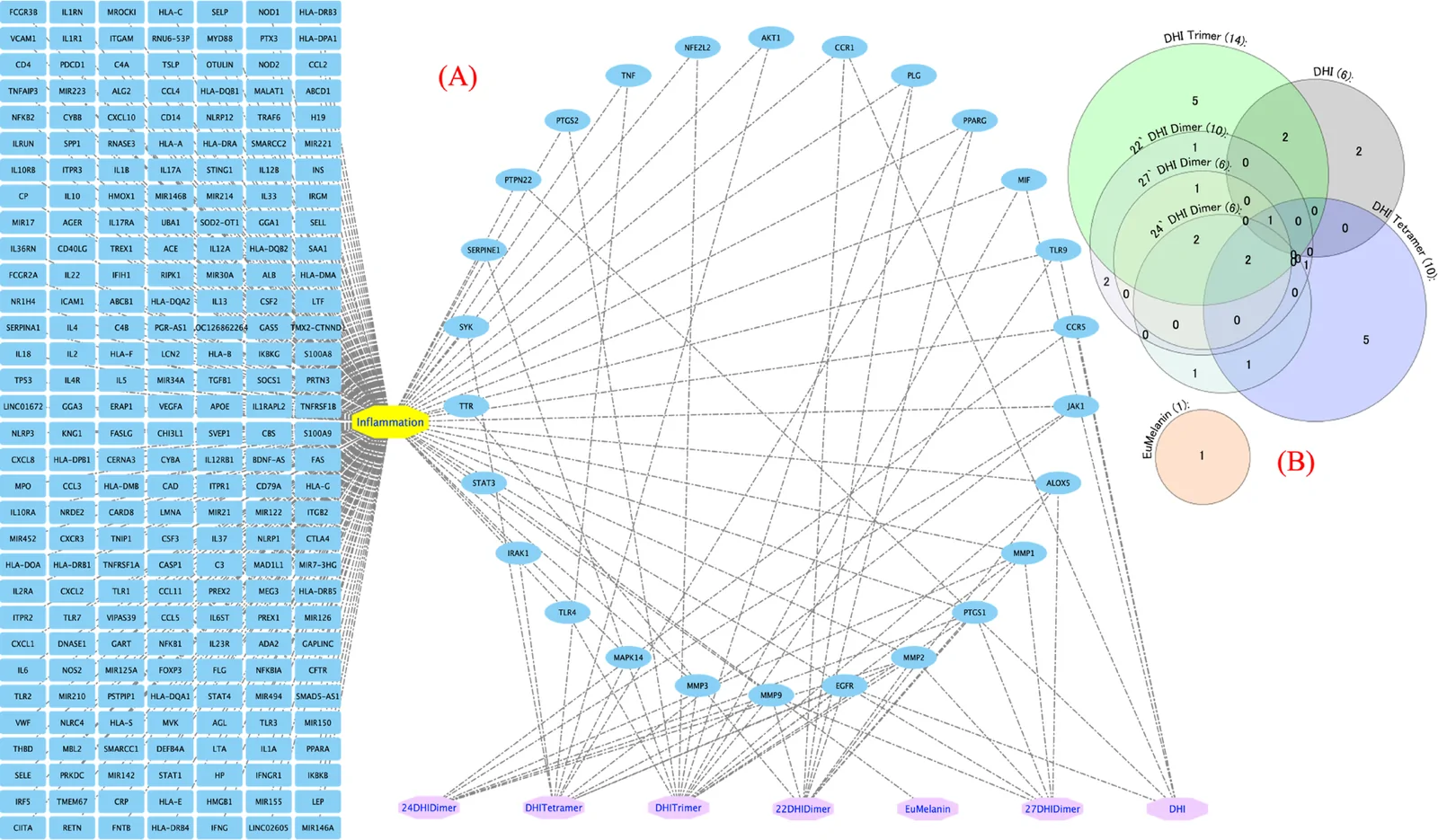
Network pharmacology of the 7-DHI-based fragments and inflammatory biomarkers (**A**). Venn Diagram (**B**).

Among the best relevance-scored 250 predicted inflammatory mediators, 26 inflammatory protein targets are the only overlapped with the predicted potential targets of the 7-tested DHI-based fragments. The drug-protein interaction network pharmacology (Fig. 12A, B) reveals the involvement of these 26 overlapped inflammatory biomarkers with promising confidentiality. Prostaglandin-endoperoxide synthase-1/2 (PTGS1 and PTGS2), the main targets for nonsteroidal anti-inflammatory drugs ‘NSAIDs’, are catalyzing the committed-step in prostaglandin bio-synthesis^93,94^, and considered among these 26 overlapped inflammatory targets. The constitutively expressed COX-1/PTGS1^94–96^ might be strongly inhibited by 4 of the 7-tested DHI-based fragments, including 2,2′ DHI-dimer, 2,4′ DHI-dimer, 2,7′ DHI-dimer, and DHI-trimer. Whereas, the inflammatory and pathologically induced COX-2^94,96^ might only be inhibited by DHI-trimer.

#### Molecular docking analysis

**Fig. 13:**
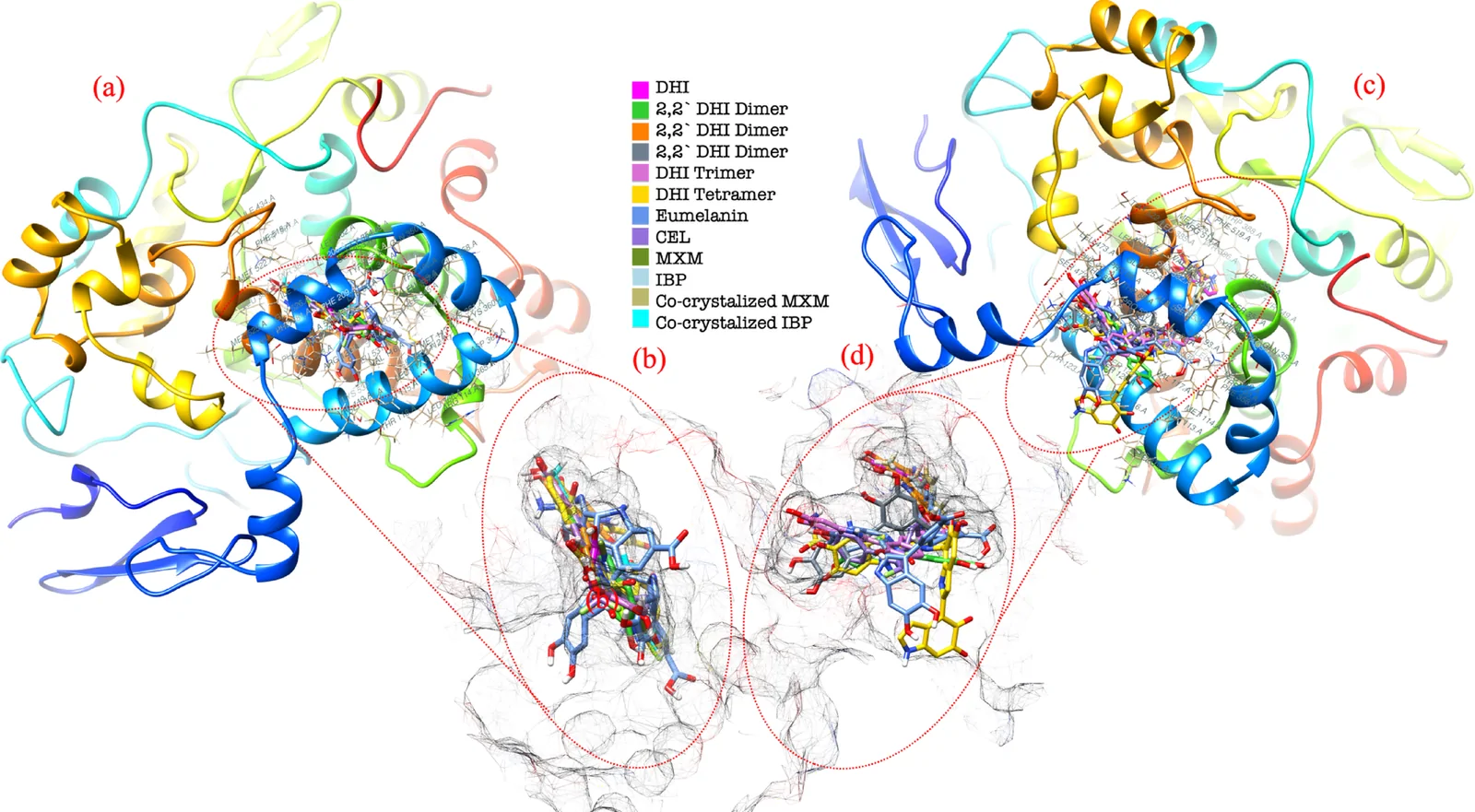
3D structural presentation of the interaction of DHI mono/oligomeric fragments on the active site of both COX-1 [PDBID: 4O1Z] and COX-2 [PDB ID: 4PH9]. Orientation of all superimposed docked conformes on the active site pocket of COX-1 chain A [4O1ZchA] and COX-2 chain A [4PH9ChA] expressed in ribbon (**a** and **c**, respectively) and mesh hydrophobic surface (**b** and **d**, respectively) presentations. MXM: Meloxicam; IBP: Ibuprofen; CEL: Celecoxib.

**Fig. 14:**
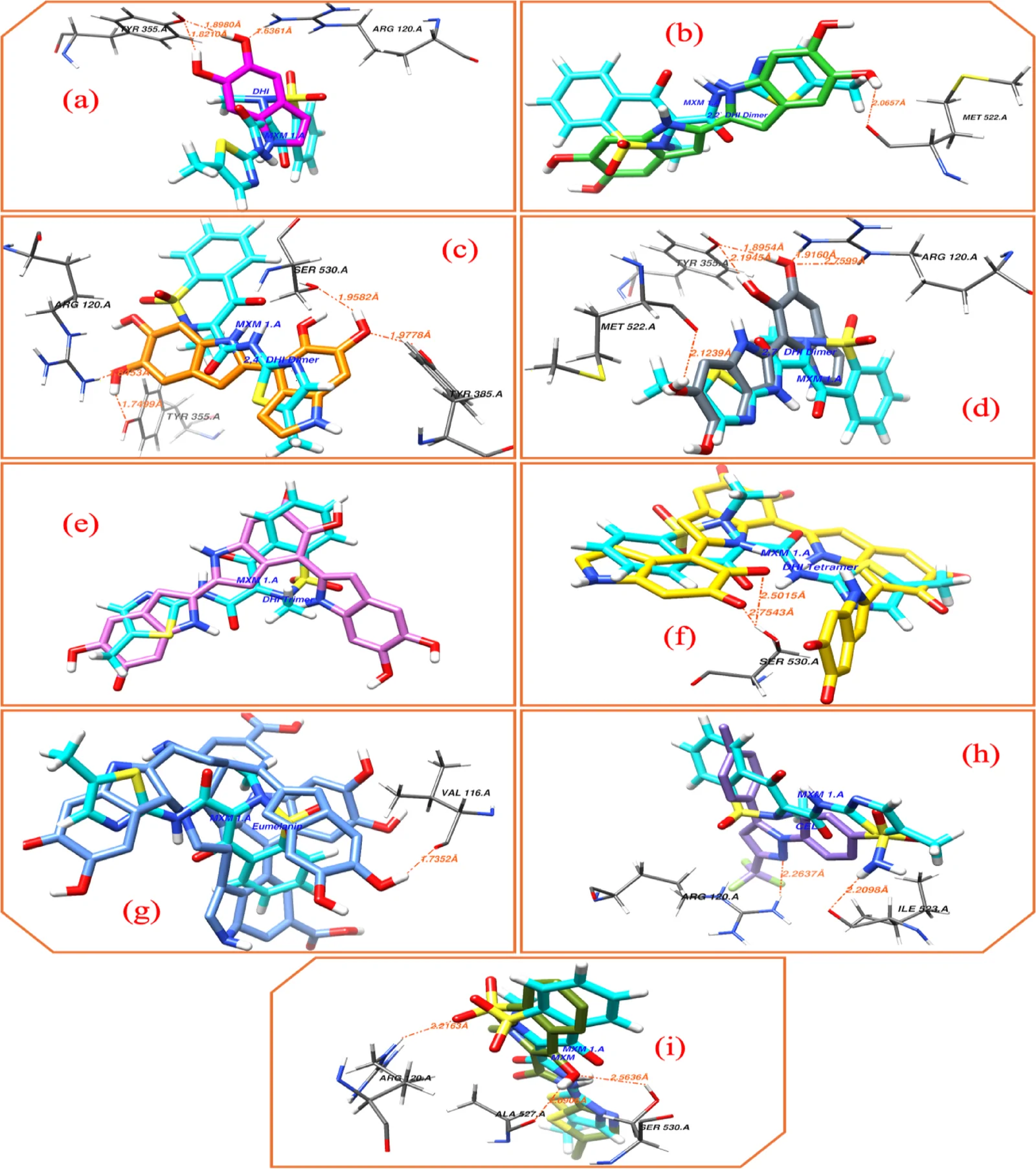
3D presentation of the interaction poses of mono/oligomeric DHI fragments with COX-1 [PDB ID: 4O1Z]. DHI (**a**), 2,2′ DHI-dimer (**b**), 2,4′ DHI-dimer (**c**), 2,7′ DHI-dimer (**d**), DHI-Trimer (**e**), DHI Tetramer (**f**), Polycyclic Eumelanin (**g**), Celecoxib (CEL) (**h**), and Meloxicam (MXM) (**i**) are colored in magenta, Lime green, Orange, Slate grey, Orchid, Gold, Cornflower-blue, Medium-Purple, and Olive-drab, respectively. The COX-1 co-crystalized ligand, MXM, is colored in cyan. H-bonds are presented as dashed orange lines.

**Fig. 15:**
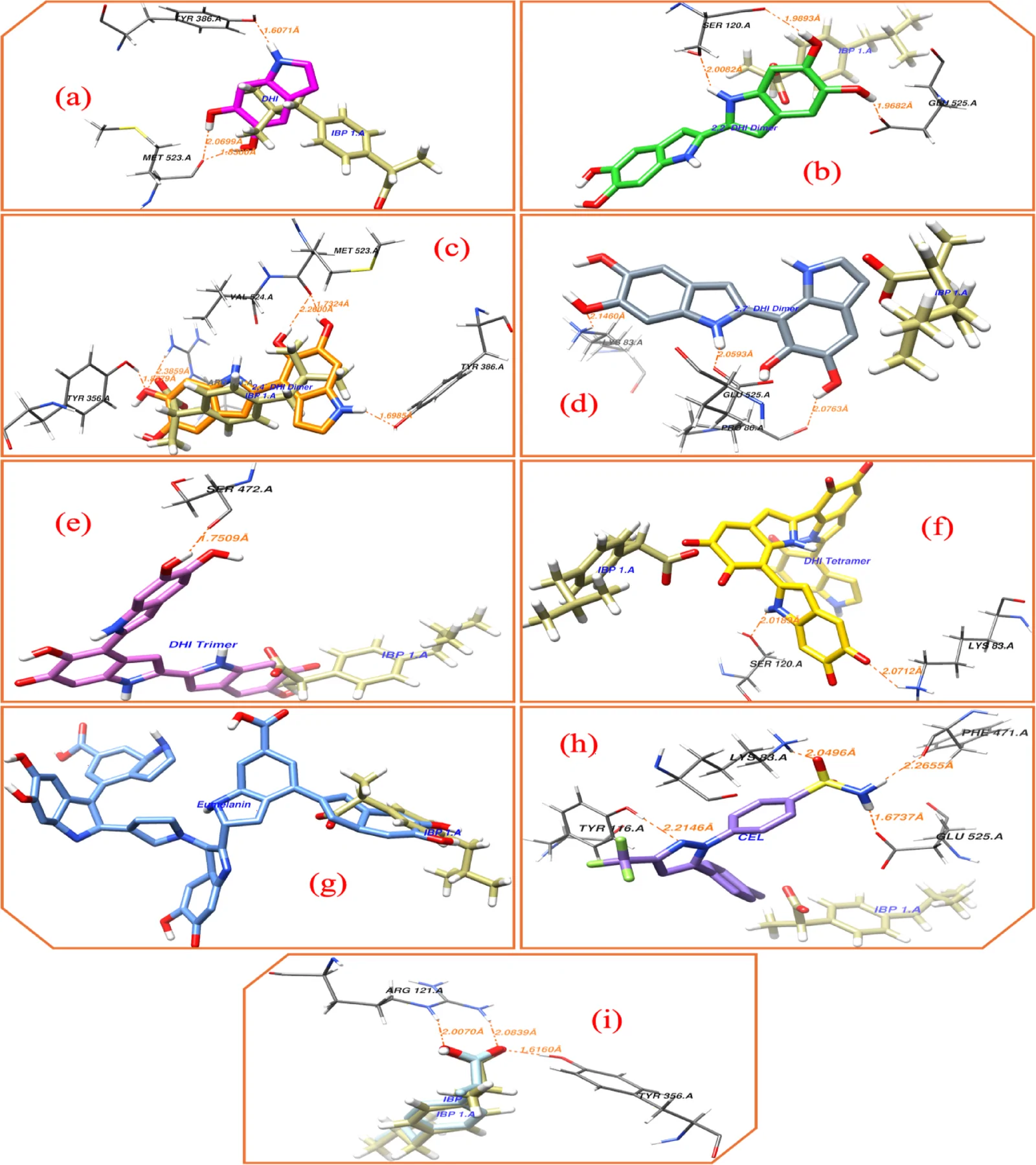
3D presentation of the interaction poses of mono/oligomeric DHI fragments with COX-2 [PDB ID: 4PH9]. DHI (**a**), 2,2′ DHI-dimer (**b**), 2,4′ DHI-dimer (**c**), 2,7′ DHI-dimer (**d**), DHI-Trimer (**e**), DHI Tetramer (**f**), Polycyclic Eumelanin (**g**), Celecoxib (CEL) (**h**), and Ibuprofen (IBP) (**i**) are colored in magenta, Lime-green, Orange, Slate-grey, Orchid, Gold, Cornflower-blue, Medium-Purplr, and Olive-drab, respectively. The COX-2 co-crystalized ligand, IBP, is colored in dark khaki. H-bonds are presented as dashed orange lines.

In silico molecular docking analysis of eumelanin mono/oligomeric fragments was performed to postulate the plausible binding modes of their anti-inflammatory interactions in both in vivo and in vitro kinetics of COX-1 and COX-2. X-ray crystal structure with the best resolution (r)-value of COX-1 [PDBID: 4O1Z, r: 2.40°A] and COX-2 [PDBID: 4PH9, r: 1.81°A] was selected to retrieve from the Protein Data Bank ‘PDB’. The orientation of all docked conformers on both COX-1 and COX-2 binding pockets is postulated in Fig. 13. The best lowest binding energies (ΔG) of the docked conformers’ poses are summarized in Table 6. Amino acid residues involved in hydrophobic and hydrophilic H-bonding interactions with their bond lengths are 2D and 3D sketched in Figs. 14 and 15, Supplementary Figs. 5 and 6.

**Table 6:** Binding energy (ΔG) in Kcal/mol of the docked conformers on COX-1 [PDB ID: 4O1Z] and COX-2 [PDB ID: 4PH9]:

	COX-1 (PDB ID: 4O1Z)	COX-2 (PDB ID: 4PH9)
	Binding energy (ΔG) (Kcal/mol)	
*Eumelanin-fragments*		
DHI	− 3.74	− 3.92
2,2′ DHI-dimer	− 4.71	− 3.97
2,4′ DHI-dimer	− 5.93	− 5.27
2,7′ DHI-dimer	− 5.89	− 4.69
DHI-trimer	− 1.95	− 3.14
DHI-tetramer	+ 21.82	− 4.4
Polycyclic eumelanin	+ 105.98	− 2.1
*Validation (Re-docking)*		
MXM	− 7.95	…….
IBP	…….	− 6.56
*Standard*		
CEL	− 5.85	− 6.38

DHI: 5,6-dihydroxy-indole; 2,2′ DHI-dimer: 5,6,5′,6′-tetrahydroxy-2,2′-biindolyl; 2,4′ DHI-dimer: 5,6,5′,6′-tetrahydroxy-2,4′-biindolyl; 2,7′ DHI-dimer: 5,6,5′,6′-tetrahydroxy-2,7′-biindolyl; MXM: Meloxicam; IBP: Ibuprofen; CEL: Celecoxib.

Results of in silico molecular docking simulation inferred that the docked conformers of the three DHI-dimeric fragments bind well with the COX-1 active binding site compared to the COX-2 one. While the other mono/oligomeric fragments show higher affinity to the binding pocket of COX-2 than that of COX-1. Among the 7-tested eumelanin DHI-based fragments, 2,4′ DHI-dimer exhibits the most excellent COX-1 and COX-2 active site binding affinity with binding energy (ΔG) values of − 5.93 kcal/mol and − 5.27 kcal/mol, respectively.

Meloxicam (MXM), used for redocking validation on the co-crystalized COX-1 [PDBID: 4O1Z] active site, shows the highest binding affinity (ΔG) of − 7.95 kcal/mol, followed by the dimeric fragment 2,4′ DHI-dimer (− 5.93 kcal/mol), 2,7′ DHI-dimer (− 5.89 kcal/mol), and then the standard Celecoxib (CEL) (− 5.85 kcal/mol). While, the polymeric fragments of Polycyclic eumelanin (+ 105.98 kcal/mol), DHI-tetramer (+ 21.82 kcal/mol), and DHI-trimer (− 1.95 kcal/mol) reflect the unstable lowest binding affinity to COX-1 active site. These unusually high positive binding energies observed for the tetramer and polycyclic eumelanin (+ 21.82 kcal/mol and + 105.98 kcal/mol, respectively) are likely attributable to their large molecular size, highly rigid polyaromatic structure, and extensive steric interactions within the receptor binding pocket, resulting in energetically unfavorable docking poses and poor binding affinities. On the other hand, the re-docked ibuprofen (IBP) and the standard drug celecoxib (CEL), have the highest COX-2 [PDB ID: 4PH9] active site binding affinity (ΔG) of − 6.56 kcal/mol and − 6.38 kcal/mol, respectively. Also, 2,4′ DHI-dimer would be the most effective among the tested DHI mono/oligomeric fragments, with a binding affinity on the COX-2-active site of − 5.27 kcal/mol. Similar to COX-1, unstable lowest binding affinities on the COX-2 active site were also shown by the DHI-polymeric fragments of polycyclic eumelanin (− 2.1 kcal/mol), DHI-trimer (− 3.14 kcal/mol), and DHI-tetramer (− 4.4 kcal/mol), respectively.

The two oxygens (O1 and O2) of the di-hydroxylated benzene ring of DHI indole moiety are H-bonded to the Tyr-355 oxygen (1.821 and 1.898°A) and the guanidino nitrogen of Arg-120 (1.636°A) within the COX-1 binding pocket (Fig. 14a, Supplementary Fig. 5a). While they form H-bond with Met-523 oxygen (2.070 and 1.830°A) in the COX-2 binding pocket that also supports an additional H-bond between Tyr-386 oxygen and the nitrogen of the DHI indole moiety’s 5-membered pyrrole ring with a bond distance of 1.607°A (Fig. 15a, Supplementary Fig. 6a). The binding mode of 2, 2′ DHI-dimer affords H-bond formation with COX-1 Met-522 (2.066°A) (Fig. 14b), and COX-2 Ser-120 (2.008 and 1.989°A) and Glu-525 (1.968°A) (Fig. 15b). Additionally, two van der Waals π–cation interactions are non-covalently generated between the nitrogenous cation of COX-1 Arg-120 (Supplementary Fig. 5b) or COX-2 Arg-121 (Supplementary Fig. 6b) and both π-aromatic pyrrole and benzene rings of its indole moiety. Another hydrophobic non-covalent π–π stacking intermolecular interaction of both π-aromatic rings is generated with COX-2 Arg-121 and Tyr-116 (Supplementary Fig. 6b). 2,4′ DHI-dimer forms H-bonds with COX-1 binding residues of Arg-120, Tyr-355, Tyr-385, and Ser-530 with bond distances of 1.845, 1.750, 1.978, and 1.958°A, respectively (Fig. 14c, Supplementary Fig. 5c). Similarly, it is H-bonded to COX-2 binding residues of Arg-121 (2.386°A), Tyr-356 (1.878°A), Tyr-386 (1.698°A), and Met-523 (1.732 and 2.260°A) (Fig. 15c, Supplementary Fig. 6c). Non-covalent π–π stacking is also seen between COX-2 Tyr-356 residue and its indole moiety’s benzene ring (Supplementary Fig. 6c). COX-1 active site residues, including Arg-120 (2.760, and 1.916°A), Tyr-355 (2.194, and 1.895°A), and Met-522 (2.124°A), are involved in the hydrophilic H-bonding interaction with 2,7′ DHI-dimer (Fig. 14d, Supplementary Fig. 5d), while those of COX-2 active site are including Lys-83, Glu-525, and Pro-86 with bond distances of 2.146, 2.059, and 2.076°A, respectively (Fig. 15d, Supplementary Fig. 6d). The π– aromatic benzene ring of 2,7′ DHI-dimer indole moiety is involved in the π–cation interaction with the nitrogenous cation of COX-2 Arg-121 (Supplementary Fig. 6d). The DHI-trimer exhibits hydrophilic H-bond formation with a bond distance of 1.751°A to the Ser-472 residue within the COX-2 binding pocket (Fig. 15e, Supplementary Fig. 6e). It also displays hydrophobic non-covalent π–cation and π–π stacking interaction with COX-2 Tyr-356 and Lys-83, respectively (Supplementary Fig. 6e). COX-1 Ser-530 (Fig. 14f) and COX-2 Lys-83 and Ser-120 residues (Fig. 15f) can all be H-bonded by the DHI-tetramer. A non-covalent π–π stacking to COX-1 Tyr-385 (Supplementary Fig. 5f.) and COX-2 Tyr-116 (Supplementary Fig. 6f.) residues is facilitated by the hydrophobic interaction of its π–aromatic pyrrole ring. Furthermore, it establishes a π–cation contact with the COX-2 Arg-121 residue’s nitrogenous atom (Fig. 15f). Polycyclic eumelanin fragment (O44) may sterically interfere with the side chain of Val-116 with the closest distance of 1.735 Å (Fig. 14g, Supplementary Fig. 5g).

Celecoxib (CEL) is a known COX inhibitor that is used as an anti-inflammatory reference drug. It can be H-bonded to Cox-1 residues of Arg-120 and Ile-523 with bond distances of 2.264 and 2.210 Å (Fig. 14h, Supplementary Fig. 5h). While it binds COX-2 residues of Lys-83, Tyr-116, Phe-471, and Glu-525 are H-bonded with bond distances of 2.050, 2.215, 2.266, and 1.674, respectively (Fig. 15h, Supplementary Fig. 6h). Moreover, Celecoxib π–aromatic rings engage non-covalently through π–cation and π–π stacking with the COX-2 residues of Arg-121 and Tyr-116, respectively (Supplementary Fig. 6h).

Other well-known COX inhibitors including meloxicam (MXM) and ibuprofen (IBP), that were re-docked on their respective co-crystalized COX-1 [PDBID: 4O1z], and COX-2 [PDBID: 4PH9] macromolecules, respectively. Sharing the approximate identical 3D orientation of the re-docked MXM (Fig. 14i, RMSD-value of 3.606°A) and IBP (Fig. 15i, RMSD-value of 1.283°A) on their co-crystallized macromolecules (COX-1 and COX-2, respectively), confirms the validated docking method used. Meloxicam shows H-bond formation with COX-1 residues of Arg-120, Ser-530, and the backbone oxygen of Ala-527 with bond distances of 2.216, 2.564, and 2.091°A, respectively (Fig. 15i, Supplementary Fig. 5i). While ibuprofen (Fig. 15i, Supplementary Fig. 6i) shows H-bond formation with COX-2 residues of Arg-121 (2.007 and 2.084°A), and Tyr-356 (1.616°A). This maintenance of the redocked poses MXM and IBP correctly localized, producing the essential H-bond interactions within the binding pockets of COX-1 and COX-2, respectively. Therefore, the docking protocol was considered sufficiently reliable for subsequent comparative docking studies.

The binding modes of interaction of the docked conformers and cyclooxygenases’ channels are quite variable. Two of the possible binding modes to cyclooxygenases’ active site are commonly observed in the substrate arachidonic acid (AA) and some of the NSAIDs interaction. The first binding mode, an AA productive conformation, involves the H-bond formation of the AA carboxylated group to the active site residues of Arg-120 and Tyr-355^93–97^. Similarly, the majority of the classic NSAIDs, including indomethacin and profens, typically bind the cyclooxygenase channel via ionic interaction between their carboxylate group and the Arg-120 and H-bonding interaction with the Tyr-355^94^. Both docked conformers of DHI and 2,7′ DHI-dimer fragments occupy quite a similar binding mode involving COX-1 Arg-120 and Tyr-355 residues. The second binding mode is an AA non-productive conformation, where the AA carboxylate group binds with the catalytic Tyr-385 as well as Ser-530 residues^98^. It has been reported that diclofenac, an NSAID, can also bind the cyclooxygenase channel via the same residues^99^. DHI docked conformer displays the corresponding interaction in COX-2 Tyr-386 and Met-523 residues. The most promising binding mode of interaction is observed via the docked conformer of 2,4′ DHI-dimer, where it binds in the cyclooxygenase channel with both of the previously mentioned productive and non-productive binding modes of interaction, involving the four residues of COX-1 Arg-120, Tyr-355, Tyr385, and Ser-530 or COX-2 Arg-121, Tyr-356, Tyr386, and Met-523. This mode of interaction was previously reported via a class of NSAIDs called oxicams like meloxicam and piroxicam^74^.

## Conclusion

The current study successfully demonstrated the bioreactor-based optimization of EuM-NPs production by *Streptomyces glaucescens* strain NEAE-H in a 7-L under controlled fermentation conditions. Compared with the previously reported shake-flask process^22^, the optimized bioreactor system significantly reduced the cultivation time required for EuM-NPs production from 144 to 22 h while maintaining a comparable production yield. Comprehensive physicochemical characterization confirmed the formation of stable EuM-NPs with nanoscale dimensions, favorable colloidal and thermal stability, and characteristic structural features, as verified by TEM, DLS, ζ-potential, EDX, TGA, XRD, and LC–MS/MS analyses. Biological investigations revealed significant anticancer activity against Ehrlich ascites carcinoma and solid tumors, as evidenced by marked tumor growth inhibition, prolonged survival, and a reduction in tumor weight of up to 78%. Furthermore, EuM-NPs exerted potent anti-inflammatory activity, evidenced by 62.62% inhibition of carrageenan-induced paw edema and selective inhibition of COX-2. In silico analysis proposed the most realistic binding modes of interaction of the seven possible eumelanin DHI-redox fragments on both COX-1 and COX-2 activities. 2,4′-DHI-dimer was supposed to have the best binding affinity to both anti-inflammatory cyclooxygenases. There is no evidence about the exact mechanism of EuM-NPs at the molecular level. However, our *in-silico* results support the concept that its cellular degradation into DHI-mono/oligomeric fragments might enhance its bioactivity. These findings highlight the potential of bioreactor-optimized EuM-NPs as sustainable and multifunctional nanomaterials for future biomedical and pharmaceutical applications.

### Limitations

A limitation of this study is that molecular docking simulation was performed using seven DHI derivatives as representative structural units of eumelanin rather than intact EuM-NPs, since their heterogeneous polymeric structure without a uniquely defined three-dimensional structure, conventional docking approaches cannot accurately model the complete nanoparticles. Accordingly, the docking results should be regarded as indicative of potential interactions of eumelanin-derived structural units with the target proteins and not as a direct representation of nanoparticles–protein binding.

## Supplementary Information

Below is the link to the electronic supplementary material.


Supplementary Material 1


## Data Availability

All data generated or analyzed during this study are included in this article.
