# Supplementary material for: Bioreactor-based production of eumelanin nanoparticles by Streptomyces glaucescens NEAE-H: physicochemical characterization, anticancer and anti-inflammatory activities, and in silico analysis
Source: Sci Rep. 2026 Sep 1;16:27380. doi: 10.1038/s41598-026-65150-y (PMC13534705; doi:10.1038/s41598-026-65150-y)
Supplement: Supplementary file 1 [file 41598_2026_65150_MOESM1_ESM.docx]

**Bioreactor-based production of eumelanin nanoparticles by *Streptomyces glaucescens* NEAE-H: physicochemical characterization, anticancer and anti-inflammatory activities**

**^1^Noura El-Ahmady El-Naggar*,** **^1^Sara M. El-Ewasy,** **^1^Nancy M. El-Shweihy, ^2^Manar G. Helal, ^3^Eman M. Sarhan**

**^1^**Department of Bioprocess Development, Genetic Engineering and Biotechnology Research Institute, City of Scientific Research and Technological Applications, New Borg El- Arab City, 21934, Alexandria, Egypt.

**^2^**Biochemistry Department, Faculty of Science, Alexandria University, Alexandria, Egypt.

^3^Department of Pharmacology and Toxicology, Faculty of Pharmacy, Mansoura University, Mansoura 35516, Egypt.

*To whom correspondence should be addressed.

**Dr. Noura El-Ahmady Ali El-Naggar**

**Address:**

Bioprocess Development Department,

Genetic Engineering and Biotechnology Research Institute,

City of Scientific Research and Technological Applications,

New Borg El- Arab City, 21934, Alexandria, Egypt

**Tel:** (002)01003738444

**Fax:** (002)03 4593423

**E-mail:** nouraalahmady@yahoo.com

**Supplementary Table 1.** Solubility of purified melanin.

| **Treatment** | **Solubility** |
| --- | --- |
| Water, NaOH(1N), KOH(1N), Na_2_CO_3_ (1N), NH_4_OH (1N), Methanol, DIMSO | Soluble |
| Ethanol, Acetone, Acetone, Chloroform, Ethyl acetate, Benzene, Xylene, Hexane, Acetonitrile, Acetic acid, Petroleum ether, HCl (1N) | Insoluble |

|  **(A)**;  **(B)**;  **(C)** **Supplementary Figure 1.** Stability of EuM-NPs produced by *Streptomyces glaucescens* NEAE-H.  **(A)** | |
| --- | --- |
|   **(B)**  **Mix** |   **C Ka**  **(C)** |
|   **(D)**  **N Ka** |   **(E)**  **O Ka** |

**Supplementary Figure 2.** **A)** EDX analysis and **B-E)** elemental mapping analysis showing the distribution of the major constituent elements detected in the EuM-NPs.

**Supplementary Figure 3.** 2D structure of the black chromogenic 5,6-dihydroxy-indole (DHI)-based eumelanin mono/oligo/polymeric precursors. (a) DHI: 5,6-dihydroxy-indole; (b) 2,2`-biindolyl (2,2` DHI-dimer); (c) 2,4`-biindolyl (2,4` DHI-dimer); (d) 2,7`-biindolyl (2,7` DHI-dimer) (e) Linear DHI-trimer; (f) macrocyclic DHI-tetramer; (g) Polycyclic DHI-pentamer eumelanin.

**Supplementary Figure 4.** BOILED-Egg Model of eumelanin and its mono/oligo/polymeric precursors. *BBR: Blood Brain Barrier; HIA: Human Intestinal Absorption; PGP+/-: P-glycoprotein.*

**Supplementary Figure 5.** 2D presentation of the interaction poses of mono/oligomeric DHI fragments with COX-1 [PDB ID: 4O1Z]. DHI (a), 2,2` DHI-dimer (b), 2,4` DHI-dimer (c), 2,7` DHI-dimer (d), DHI-Trimer (e), DHI Tetramer (f), Polycylic Eumelanin (g), Celecoxib (CEL) (h), and Meloxicam (MXM) (i).

**Supplementary Figure 6.** 2D presentation of the interaction poses of mono/oligomeric DHI fragments with COX-2 [PDB ID: 4PH9]. DHI (a), 2,2` DHI-dimer (b), 2,4` DHI-dimer (c), 2,7` DHI-dimer (d), DHI-Trimer (e), DHI Tetramer (f), Polycylic Eumelanin (g), Celecoxib (CEL) (h), and Ibuprofen (IBP) (i).
